# Repeatable Photoactive Stent-Based Catheter to Enhance Therapeutic Efficacy for Esophageal Carcinoma

**DOI:** 10.34133/bmr.0274

**Published:** 2025-10-30

**Authors:** Seung Jin Eo, Hyeonseung Lee, Ji Won Kim, Song Hee Kim, Dong-Sung Won, Yubeen Park, Kun Na, Do Hoon Kim, Jung-Hoon Park

**Affiliations:** ^1^ Department of Convergence Medicine, Asan Medical Center, University of Ulsan College of Medicine, Seoul 05505, Republic of Korea.; ^2^ Biomedical Engineering Research Center, Asan Institute for Life Sciences, Asan Medical Center, Seoul 05505, Republic of Korea.; ^3^ Department of Biotechnology, Department of Biomedical-Chemical Engineering, The Catholic University of Korea, Bucheon-si, Gyeonggi-do 14662, Republic of Korea.; ^4^ Department of Gastroenterology, Asan Medical Center, University of Ulsan College of Medicine, Seoul 05505, Republic of Korea.

## Abstract

Localized photodynamic therapy (PDT) using a photoactive stent-based catheter involves the direct delivery of reactive oxygen species to the mucosa in esophageal carcinoma; however, the damaged mucosa recovers within 2 to 4 weeks, which considerably limits the clinical application of PDT. Here, we used aluminum(III) phthalocyanine chloride tetrasulfonic acid (AlPcS4) as a photosensitizer due to its excellent photochemical durability, low photobleaching, and high quantum yield and investigated whether repeated and periodic PDT via an AlPcS4-embedded stent-based catheter can provide sustained therapeutic efficacy. AlPcS4 was uniformly embedded in silicone membranes via coordination bonding to form a photoactive stent-based catheter. The membrane demonstrated excellent photostability and consistent singlet oxygen generation under near-infrared irradiation, as evidenced by a 67.2% decrease in 9,10-dimethylanthracene fluorescence intensity, even after 90 J cm^−2^ irradiation, markedly superior to methylene blue (15.3%) and chlorin e6 (30.9%). Repeated PDT effectively enhanced cell death rates in KYSE-70 cells. In the xenograft model, MRI-based volumetric analysis showed that the tumor volume change in the thrice-PDT group (57.40% ± 9.26%) was significantly lower than those in the control (212.07% ± 38.44%, *P* < 0.001) and once-PDT groups (130.77% ± 11.25%, *P* = 0.018), accompanied by apoptotic and necrotic tumor destruction. Repeated PDT at 1-week intervals was technically successful in the porcine esophagus, leading to progressive mucosal injury, luminal narrowing, and apoptosis, while demonstrating sustained therapeutic efficacy. Thus, the minimally invasive repeatable photoactive stent-based catheter may be an effective and safe approach for treating esophageal carcinoma.

## Introduction

Esophageal carcinoma is the 11th most commonly diagnosed cancer and the 5th leading cause of cancer-related deaths worldwide. Progression of esophageal carcinoma may cause severe dysphagia, malnutrition, cachexia, and aspiration of food, which substantially impact the quality of life of the patients [1]. Over the past decades, self-expanding metal stents (SEMSs) have been considered the gold standard for palliative care and an effective therapeutic option for progressive esophageal carcinoma [2,3]. However, recurrent dysphagia resulting from stent-related complications remains a major obstacle to the success of palliative therapy using SEMSs. Recently, highly functionalized SEMSs have been actively developed and investigated as localized and direct ablation modalities to reduce tumor volume via radio-frequency ablation and irreversible electroporation [4–8]. However, the esophagus, being anatomically surrounded by critical structures including the heart, lungs, and trachea, remains an area of serious concern due to the risk of thermal injury to major organs or induction of electrically induced arrhythmias [9,10]. Therefore, safer alternative therapeutic strategies for esophageal carcinoma should be developed.

Photodynamic therapy (PDT) has been used for decades to treat diseased esophageal mucosa in the setting of squamous dysplasia and carcinoma [11]. PDT is a minimally invasive therapeutic modality that relies on light-induced activation of a photosensitizer (PS) to produce reactive oxygen species (ROS), leading to targeted cellular destruction. In clinical practice for esophageal carcinoma, the PS is administered via an oral or intravenous route and it localizes to target tumor cells; then, light of a specific wavelength activates the PS, leading to selective cell necrosis and apoptosis via oxidative stress. The advantages of PDT include noninvasiveness, high selectivity, and minimal side effects or damage to cells away from the irradiated site. However, its clinical applications have been limited due to insufficient accumulation of the PS in tumors and the shallow tissue penetration depth of the light source [12]. In addition, another major drawback is that patients receiving a systemically administered PS must avoid sunlight and strong artificial light for weeks to prevent photosensitivity, which can cause considerable discomfort.

To address these limitations, a PDT using a PS-embedded membrane-covered stent was developed [13–16]. The PS-embedded covering membrane of the stent successfully generated sufficient ROS under laser irradiation, enabling localized PDT directly to the tumoral bile duct or esophagus without systemic PS administration. Furthermore, recent advancements have allowed the stents to be catheterized, enabling immediate removal of the stent-based catheter system after PDT and even delivery of laser irradiation to the surface of the placed stent areas by passing the cylindrical fiber through the transparent inner tube of the catheter system [17,18]. Localized PDT using the stent-based catheter successfully induced significant mucosal and submucosal damages without procedure-related complications in a minimally invasive procedure. However, this therapeutic effect was maintained for only 1 to 2 weeks after the endoluminal PDT procedure, and the mucosa gradually recovered from the damage in 2 to 4 weeks. Short-term effects and rapid tissue recovery due to relatively low depth penetration are major limitations for clinical application, suggesting that a repeated or periodic PDT approach may be required to sustain therapeutic efficacy.

Aluminum(III) phthalocyanine chloride tetrasulfonic acid (AlPcS4) has been extensively investigated as a second-generation PS due to its high ROS generation efficiency and water solubility. However, when administered intravenously, its low delivery efficiency and strong binding to serum albumin limit its accumulation in tumor cells, thereby reducing its therapeutic efficacy [19,20]. The previously mentioned photoactive stent-based catheter system is a novel localized PDT strategy that overcomes the low delivery efficiency of AlPcS4 while retaining its intrinsic advantages. Furthermore, AlPcS4 has been reported to exhibit high photostability and minimal photobleaching effect, maintaining its molecular conformation and photodynamic functionality even under prolonged and repeated light irradiation [21,22]. Therefore, the AlPcS4-embedded membrane may ensure sustained and efficient ROS generation under repeated light exposure, contributing to its therapeutic effect in photodynamic applications.

**Fig. 1.**
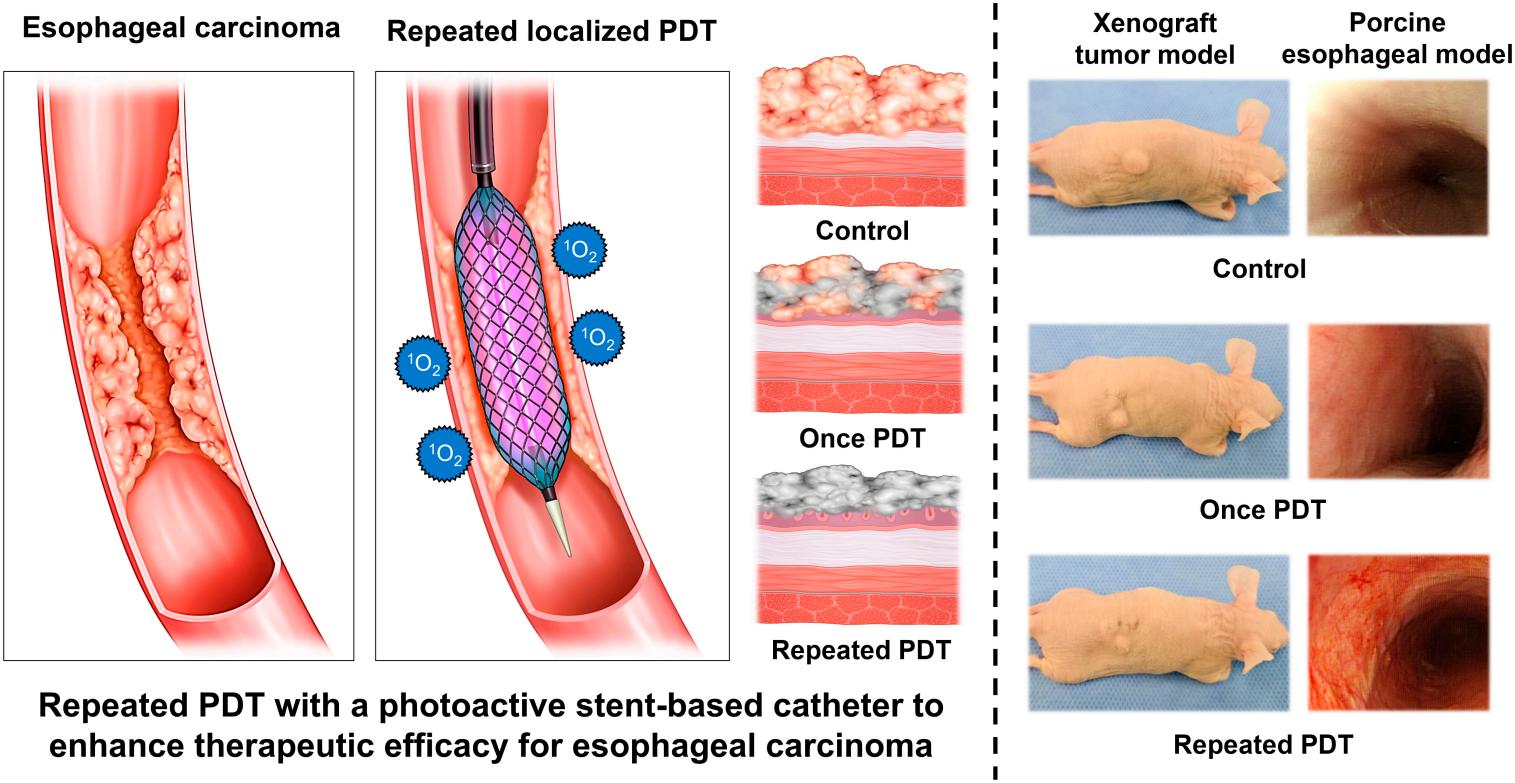
Schematic illustration of repeated and localized PDT using a photoactive stent-based catheter in xenograft tumor and porcine esophageal models. AlPcS4-based repeated PDT successfully enhanced the duration of therapeutic efficacy for esophageal carcinoma. AlPcS4, aluminum(III) phthalocyanine chloride tetrasulfonic acid; PDT, photodynamic therapy.

We have previously suggested using the AlPcS4-embedded stent-based catheter system for the local treatment of esophageal carcinoma [18]. However, much remains unknown about the clinical possibilities of repeatable PDT with the excellent photostability and minimal photobleaching feature of AlPcS4. Therefore, we hypothesized that repeated and periodic PDT using an AlPcS4-embedded stent-based catheter system can effectively enhance therapeutic efficacy against esophageal carcinoma by overcoming the depth limitation of single-session PDT via sustained and localized ROS generation. To identify the most suitable PS for repeated PDT, we compared the photoconversion efficiency and stability of methylene blue hydrate (MB), chlorin e6 (Ce6), and AlPcS4 under various conditions (long laser irradiation or high-temperature exposure). AlPcS4 was selected as the optimal PS. Fabrication of the AlPcS4-embedded membrane was confirmed using x-ray photoelectron spectroscopy (XPS). Repeated PDT using the AlPcS4-embedded membrane was assessed using trypan blue staining for qualitative measurement, and annexin V–fluorescein isothiocyanate (FITC) apoptosis staining for quantitative measurement. A xenograft tumor model was used to evaluate the anticancer effects. Finally, the efficacy and safety of repeated and periodic PDT using the AlPcS4-embedded stent-based catheter in the porcine esophagus model were examined using endoscopic, esophagographic, histological, and statistical analyses. Through this, we demonstrated that our repeated PDT strategy using the AlPcS4-embedded stent-based catheter system was durable and safe and might be beneficial for treating esophageal carcinoma (Fig. 1).

## Materials and Methods

### Materials

AlPcS4 and Ce6 were purchased from Frontier Scientific, Inc., USA; methanol was purchased from Samchun Chemical Co., Korea; xylene and dimethyl sulfoxide (DMSO) were purchased from Junsei Co., Japan; MB, 0.4% trypan blue solution, 9,10-dimethylanthracene (DMA), and Singlet Oxygen Sensor Green (SOSG) were purchased from Thermo Fisher Scientific Inc., USA; an annexin V–FITC apoptosis staining/detection kit (ab14085) was purchased from Abcam Limited, UK; silicone derivatives (MED-6640) were purchased from NuSil Technology LLC, USA; Roswell Park Memorial Institute (RPMI) 1640 medium, fetal bovine serum, penicillin–streptomycin antibiotics, and Dulbecco’s phosphate-buffered saline (DPBS) were purchased from HyClone Laboratories, USA; isoflurane (Ifran) was purchased from Hana Pharm. Co., Korea; Ki-67 antibody was purchased from Abcam; zolazepam and tiletamine (Zoletil 50) were purchased from Virbac, France; potassium chloride was purchased from Dai Han Pharm Co., Ltd., Korea; a 50-mm cylindrical laser fiber was purchased from LitePhamTech Group, Korea; a laser system (LAB671-1000MWCW400F) was purchased from JM Labtech, Korea; a 0.035-inch guidewire (Radifocus M) was purchased from Terumo, Japan; a contrast medium (Omnipaque 300) was purchased from GE Healthcare; a terminal deoxynucleotidyl transferase-mediated dUTP nick end labeling (TUNEL) kit was purchased from Millipore Co., USA; and caspase-3 was purchased from LifeSpan BioSciences Inc., USA. The stent and catheter were supplied by S&G Biotech, Korea.

### Preparation of the AlPcS4-embedded membrane and stent-based catheter

The AlPcS4-embedded membrane was fabricated as described previously [18]. Briefly, a mix of 7.1 ml of silicone, 2.49 ml of xylene, and 0.4 ml of each AlPcS4 solution (2.5 mg ml^−1^ in methanol) was mixed using a vortexer. The final coating solution corresponded to 0.1 mg ml^−1^ AlPcS4, and the loading on the film surface was 11.3 μg cm^−2^. Then, 200 μl or 2.5 ml of vortexed AlPcS4-embedded silicone solution was dispensed into polytetrafluoroethylene molds with spherical cavities of 1.0-, 1.5-, or 3.5-cm diameter, respectively. Then, the polytetrafluoroethylene molds were dried at 150 °C for 3 h. Membranes embedded with other PSs (MB and Ce6) were fabricated as mentioned above with minor modifications. The fabricated membranes’ thickness was analyzed using digital calipers (CD-20AXWW, Mitutoyo, Japan), in triplicate.

To prepare the AlPcS4-embedded stent-based catheter, the stent was fabricated using a hand-knitting technique with a single thread of a 0.206-mm-thick Ni–Ti alloy wire into a tubular diamond-cell configuration. The fabricated stent was dipped in the AlPcS4–silicone blended coating solution for 10 s followed by slow withdrawal. The stent was immediately dried in an oven at 150 °C for 3 h. A stent 24 mm in diameter and 100 mm in length was selected for catheter fabrication, which was used in the in vivo porcine study (Fig. S1). For fabricating the AlPcS4-embedded stent-based catheter, both ends of the stent were fixed to the catheter system for recapture and removal immediately after PDT. The delivery system was 18 Fr in diameter and 50 cm in usable length and comprised an insulating outer sheath and a pusher catheter with a guiding olive tip. Through the distal port of the catheter, a 50-mm cylindrical fiber could be introduced and advanced along the inner pathway to the central portion of the stent.

### Photobleaching, drying-induced degradation, and ROS generation by various PS-embedded membranes

Photobleaching experiments were conducted to identify the most photostable PS within silicone membranes under strong laser irradiation. Membranes embedded with MB, Ce6, or AlPcS4 were irradiated with a 671-nm near-infrared laser of 100 mW cm^−2^ intensity with an interval of 1,000 s (100 J cm^−2^) to 6,000 s (600 J cm^−2^). The absorbance spectra were measured in triplicate using ultraviolet–visible spectroscopy (UV-2450, Shimadzu, Kyoto, Japan).

Drying-induced degradation experiments were conducted mimicking the membrane fabricating conditions (150 °C). PS (AlPcS4, Ce6, and MB) powders were dried at 150 °C for 3 h, and aliquots were collected at 30-min intervals. MB and AlPcS4 were dissolved in distilled water and Ce6 was solubilized in DMSO such that the concentration of each was 2 μg ml^−1^. The absorbance spectra were measured as described above. Thermogravimetric analysis was conducted with each PS (AlPcS4, Ce6, and MB) powder. With a N_2_ gas atmosphere, powders were heated at 10 °C min^−1^ ramping from 30 to 300 °C, using a thermal analysis system (TGA2, Mettler Toledo, Switzerland).

ROS generation was assessed using DMA fluorescence decay and SOSG generation. DMA (2 μM) was solubilized in DMSO. A membrane 1.5 cm in diameter was placed at the bottom of the poly(methyl methacrylate) cuvette, in which 2 ml of DMA solution was dispensed. During 671-nm laser irradiation, fluorescence (excitation: 375 nm; emission: 434 nm) was measured at 50-s intervals for 5 min using a fluorescence spectrophotometer (RF-6000, Shimadzu, Kyoto, Japan) at an intensity of 100 mW cm^−2^. Control measurements under nonirradiated conditions were performed at the same time intervals while completely shielding the sample from light exposure. SOSG analysis was conducted with a similar method. Two milliliters of SOSG solution with a concentration of 5 µM was added to a film placed in a poly(methyl methacrylate) cuvette. The sample was irradiated with a 671-nm laser at an intensity of 100 mW cm^−2^ for 50 s, and the irradiation was repeated six times. For each laser irradiation, the 504-nm excitation, 525-nm emission fluorescence was measured.

### Characterizing the chemical state of the AlPcS4-embedded membrane

XPS (K-Alpha, Thermo Fisher, Massachusetts, USA) was performed to analyze the chemical composition and bonding states of the silicone membrane before and after AlPcS4 embedding. The membranes were etched using Ar ions at 500 eV for 100 s. A monochromatic Al Kα x-ray source was used at 12 kV and 3 mA. The survey scan was performed with a pass energy of 200 eV and a step size of 1 eV, while a pass energy of 40 eV and a step size of 0.1 eV were used for the detailed scan. The base pressure was 2.9 × 10^−9^ mbar, and the operating pressure was 4.8 × 10^−9^ mbar. Each elemental spectrum was processed using the CasaXPS software (CasaXPS version 2.3.26; California, USA). AlPcS4 powder was pelleted with SiO_2_ at a 5-mm thickness and analyzed in a manner similar to that described above.

### Phototoxicity of repeated PDT using the AlPcS4-embedded membrane

Phototoxicity assays were performed based on previous studies with minor modifications [18,23]. Human esophageal squamous cell carcinoma KYSE-70 cells (European Collection of Cell Cultures, UK) were cultured in RPMI 1640 medium supplemented with 10% fetal bovine serum and 1% penicillin–streptomycin at 37 °C in a humidified incubator with 5% CO_2_. Trypsinized KYSE-70 cells (3 × 10^5^ cells/well) were seeded into 12-well cell culture plates and incubated for 24 h under the same conditions. After washing with DPBS and removing the residual solution, AlPcS4-embedded membranes were placed in each well. Then, PDT was performed by irradiating the membranes with a near-infrared laser at 50 mW cm^−2^ for 60 s (3 J cm^−2^). DPBS was added, the AlPcS4-embedded membrane and solvent were removed, and the cells were incubated in a fresh RPMI 1640 medium. This procedure was performed at 0 h for the once-PDT group, at both 0 and 12 h for the twice-PDT group, and at 0, 12, and 24 h for the thrice-PDT group. In the sham control group, the membrane was incubated for 60 s without light exposure at each timepoint. After the last PDT procedure, cells were incubated for 15 min with 1 ml of 0.4% trypan blue staining solution. After washing twice with DPBS, the trypan blue-stained dead cells were examined under an optical microscope at ×4 magnification (ICX40, Ningbo Sunny Instruments Co., Ltd., Zhejiang, China).

For analyzing apoptosis and necrosis, KYSE-70 cells were seeded at a density of 6 × 10^5^ cells/well into 6-well cell culture plates. AlPcS4-embedded membranes of 3.5-cm diameter were used in this procedure. The same laser irradiation procedure was applied as described above. Annexin V–FITC apoptosis detection was performed using a commercial kit (manufacturer’s instructions), and stained cells were analyzed using a flow cytometer (BD FACS Canto II, Becton Dickinson, New Jersey, USA).

### Repeated PDT using the AlPcS4-embedded membrane on a xenograft tumor model

The xenograft study was approved by the Institutional Animal Care and Use Committee of the Asan Institute for Life Sciences (#2024-40-194), and it conformed to the guidelines of the USA National Institutes of Health for handling laboratory animals. The xenograft tumor model was used to evaluate the tumor regression and cell death effects. In total, 12 male BALB/c nude mice (JABIO, Suwon, Korea) were enrolled. All mice were housed in a breeding environment that maintained a 12-h night-and-day cycle at a temperature of 20 to 24 °C and humidity of 44.5% to 51.8%. At 6 weeks of age, 5 × 10^6^ KYSE-70 cells suspended in 100 μl of PBS were subcutaneously injected into the right flank of nude mice. The tumor volume was evaluated every 3 d and was calculated using the following formula: *V* = (*W*^2^ × *L*)/2, where *W* is the tumor width and *L* is the tumor length [24]. On the 10th day after inoculation of the tumor xenografts, the mice were randomly divided into 4 groups as follows: control (−membrane, −laser), once-PDT (+membrane, +laser), twice-PDT (+membrane, ++laser), and thrice-PDT groups (+membrane, +++laser). The AlPcS4-embedded membrane (1.0 cm in diameter) was surgically placed beneath the tumor (Fig. S2) [13]. Laser irradiation at 670 nm was conducted at a power density of 50 mW cm^−2^, delivering a total energy of 100 J cm^−2^ for 2,000 s. According to a previous repeated PDT study in a xenograft tumor model, the repeated irradiation interval was determined to be 2 d [25].

### MRI monitoring

MRI was performed to analyze the changes in tumor volume after PDT treatment using a 9.4-T animal MRI system (Agilent Technologies, Santa Clara, CA, USA). The animals were anesthetized in an induction chamber of 5% isoflurane with 1:1 oxygen (2 l/min). Mice were placed on the animal bed with a mask for the maintenance dose of the anesthesia (2% isoflurane). T_2_-weighted (T_2_W) MRI images were obtained in the axial section of the tumors before the procedure and 3, 6, and 10 d after PDT treatment. A T_2_W imaging sequence with field of view = 30.0 × 30.0 mm, matrix size = 256 × 256, slice thickness = 0.8 mm, repetition time = 4,000 ms, echo time = 35.17 ms, and flip angle = 90° was used. The tumor volume was independently assessed in each magnetic resonance (MR) examination using region-of-interest-based volumetry (Fig. S3) [26]. The entire tumor region was identified and traced on the MR workstation on all T_2_W axial imaging slices throughout the tumor. A 3-dimensional region-of-interest-based volume was calculated by measuring the tumor area on each axial T_2_W MRI slice and multiplying each area by the slice thickness (0.8 mm). All tumor areas across the slices were then summed to obtain the total volume. Tumor volume change was represented as a percentage of the initial volume (day 0).

### Gross and histological examination

All mice were sacrificed after obtaining an MRI on day 10 by administering inhaled pure carbon dioxide. After fixation in 4% paraformaldehyde, histological and immunohistochemical analyses were performed to evaluate cellular morphology, inflammatory responses, and therapeutic efficacy. Hematoxylin and eosin (H&E) staining was used to assess general tissue architecture and inflammatory cell infiltrations. Ki-67 staining was conducted to evaluate tumor cell proliferation, and TUNEL staining was used to detect apoptotic cell death. Histological examination was performed using a digital slide scanner (Pannoramic 250 FLASH III; 3DHISTECH Ltd., Budapest, Hungary), and representative images were obtained using a digital microscope viewer (CaseViewer; 3DHISTECH).

### Animal study design

This study was approved by the Institutional Animal Care and Use Committee of the Asan Institute for Life Sciences (#2025-40-141), and it conformed to the guidelines of the US National Institutes of Health for the humane handling of laboratory animals. In total, 8 juvenile pigs (weight range: 31.6 to 34.9 kg; median: 33.02 kg) purchased from the International Animal Experiment Center (Pocheon, Korea) were randomly assigned to 4 groups according to the number of repetitions: control, once-PDT, twice-PDT, and thrice-PDT, with 2 pigs in each group. Based on a previous study, the repetition interval was set to 1 week [23]. Animals were supplied with food and water ad libitum at 24 ± 2 °C under a 10-h day–night cycle. All pigs were euthanized at 3 weeks after the first PDT by injecting 75 mg/kg of potassium chloride via the marginal ear vein. This study was designed to evaluate whether repeated PDT could sustain the initial mucosal injury. Accordingly, the sacrifice timepoint was set based on the first PDT, following the general approach reported in previous studies [27,28].

### PDT using the AlPcS4-embedded stent-based catheter in the porcine esophagus

The AlPcS4-embedded stent-based catheter placement and PDT procedures were performed as described previously [25]. After general anesthesia, an overtube (Guardus Overtube; STERIS, Mentor, OH) was inserted into the upper thoracic esophagus under endoscopic guidance (CF-H260AI; Olympus Inc., Tokyo, Japan). The photoactive stent-based catheter was inserted into the lower thoracic esophagus over a 0.035-inch guidewire (Radifocus M; Terumo, Tokyo, Japan) under fluoroscopic guidance (Fig. S4A). The stent portion was deployed in the lower thoracic esophagus by smoothly pulling the braided tube (Fig. S4B). The guidewire was removed, the cylindrical fiber was inserted through the distal port of the catheter, and a distal marker of cylindrical fiber was placed at the lower esophagus. Laser irradiation (wavelength, 670 nm) was performed at a power density of 1,000 mW cm^−2^ and irradiation energy of 400 J cm^−2^ using a laser system (Fig. S4C) [18]. After PDT, the expanded stent was recaptured by advancing the braided tube and the catheter was smoothly removed (Fig. S4D). This procedure was performed in the same way for an assigned number of PDT sessions. Antibiotics (gentamicin, 7 mg kg^−1^; Shin Poong Pharm Ltd., Seoul, Korea) and analgesics (Keromin [ketorolac] 1 mg kg^−1^; Hana Pharm Ltd., Seoul, Korea) were routinely administered for 3 d after every PDT procedure.

### Endoscopic and esophagographic examination

Endoscopic and esophagographic examinations were performed every week after the PDT procedure. The endoscopic examination was performed to evaluate the PDT-treated mucosal changes in the esophagus. The degrees of mucosal injuries were subjectively determined as follows: 1 = mild (mucosal edema, erythema, and hyperemia), 2 = mild to moderate (submucosal tortuous blood vessel friability, erosions, hemorrhage, blisters, pseudomembrane formation due to exudates, whitish membrane, and shallow ulcers), 3 = moderate (grade II lesions in addition to deep or circumferential lesions), 4 = moderate to severe (small or scattered areas of necrosis), and 5 = severe (extensive necrosis) [29]. Esophagography was performed to evaluate the luminal patency of the PDT-treated esophagus. The esophageal luminal diameters were evaluated using the RadiAnt DICOM viewer (version 1.1.20, Medixant Company, Poland).

### Histological examinations

Surgical exploration of the entire esophagus was performed in all pigs. The extracted tissue samples were immediately fixed in 10% neutral buffered formalin for 48 h. The PDT-treated lower esophagus was sectioned transversely. The samples were stained with H&E, Masson’s trichrome, TUNEL, and caspase-3. The average degrees of inflammatory cell infiltration, collagen deposition, TUNEL-positive staining, and caspase-3-positive staining were analyzed based on the mean values obtained from 8 points around the circumference. The degrees of inflammatory cell infiltration, collagen deposition, TUNEL-positive deposition, and caspase-3-positive deposition were subjectively determined using H&E-, Masson’s trichrome-, or immunohistochemistry-stained sections (grading: 1, mild; 2, mild to moderate; 3, moderate; 4, moderate to severe; and 5, severe). Histological analyses were performed with a digital slide scanner, and measurements were obtained with a digital microscope viewer. Histological analysis was performed by a consensus of 3 observers who were blinded to the groups.

### Statistical analysis

Data are expressed as mean ± standard deviation. As appropriate, statistical significance was analyzed using a one-way analysis of variance (ANOVA) with Tukey’s test. The significance was considered as follows: *P < 0.05, **P < 0.01, and ***P < 0.001. Statistical analyses were performed using the Prism software (GraphPad version 8.0.1; La Jolla, CA, USA) and Statistical Package for the Social Sciences software (SPSS version 27.0; IBM, Chicago, IL, USA).

## Results and Discussion

### Photobleaching, drying-induced degradation, and ROS generation by various PS-embedded membranes

**Fig. 2.**
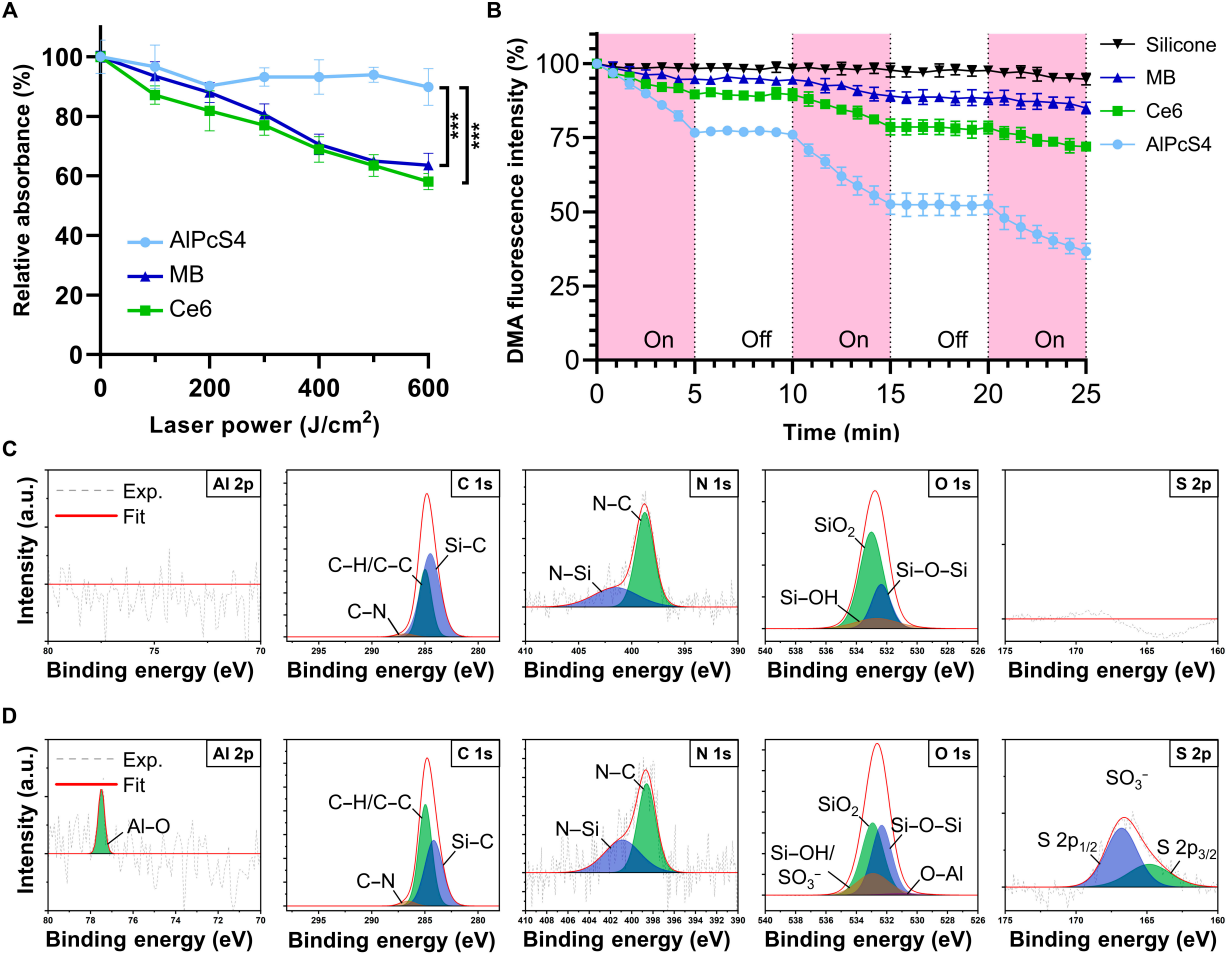
Characterization of the AlPcS4-embedded membrane. (A) Photobleaching analysis using laser irradiation of various PS-embedded membranes. (B) Analysis of ROS generation of various PS-embedded membranes using DMA based on fluorescence attenuation. The pink area indicates laser on (On), and the white area indicates laser off (Off). (C) XPS spectra of the silicone membrane at various element peaks (Al 2p, C 1s, N 1s, O 1s, and S 2p). (D) XPS spectra of the AlPcS4-embedded membrane at various element peaks (Al 2p, C 1s, N 1s, O 1s, and S 2p). AlPcS4 interacts with the silicone polymer, which induces even dispersion in the silicone membrane, further showing strong anti-photobleaching effects and ROS generation. Note: Data are presented as mean ± standard deviation. ***P < 0.001. XPS, x-ray photoelectron spectroscopy; PS, photosensitizer; ROS, reactive oxygen species; DMA, 9,10-dimethylanthracene; MB, methylene blue; Ce6, chlorin e6.

To identify the most photostable PS under repeated laser exposure, 3 PSs (MB, Ce6, and AlPcS4) were individually embedded into silicone membranes with similar thickness (~0.06 mm) and their performances were compared under prolonged and repeated irradiation (Fig. 2A and B and Figs. S5 and S6). AlPcS4, a hydrophilic second-generation PS, combines the advantages of MB (a hydrophilic but first-generation PS with a low photoconversion efficiency) and Ce6 (a hydrophobic PS with a strong photoconversion efficiency). When irradiated with up to 600 J cm^−2^ of laser, membranes embedded with MB or Ce6 showed a significant reduction in their characteristic absorbance peaks by 60%, indicating pronounced photobleaching (Fig. 2A). Such degradation of absorbance correlates with a substantial decline in photoconversion efficiency during PDT. This could compromise treatment efficiency in clinical settings employing high-intensity laser irradiation. However, the AlPcS4-embedded membrane maintained approximately 90% of its peak absorbance under the same irradiation conditions, demonstrating strong resistance to photobleaching. These findings may be explained by the following mechanism: The pronounced photobleaching of Ce6 is possibly attributed to its hydrophobic nature, which promotes aggregation within the silicone matrix. This aggregation may shift the ROS generation mechanism from a type II to a type I pathway, resulting in the production of radicals that accelerate PS reduction and photobleaching. Similarly, MB undergoes radical-mediated reduction, leading to the formation of a colorless leuco-form. In comparison, the structural stability of AlPcS4, along with its uniform dispersion in the membrane, supports a type II ROS generation pathway. This pathway minimizes self-reduction and photobleaching, thereby preserving photodynamic efficiency under intense laser irradiation [30,31].

When fabricating membrane or silicone coatings, a harsh temperature condition (150 °C for 3 h) is used for silicone polymerization and xylene drying. Absorbance changes were monitored to assess the impact of these conditions on the activity of PSs (Fig. S5A). Hydrophilic PSs, AlPcS4 and MB, exhibited a slight increase in absorbance attributed to H_2_O evaporation during the drying process. However, their absorbance remained stable for over 3 h. In contrast, Ce6 showed a significant decrease in absorbance with drying [32]. These results suggest that Ce6 is not suitable for coating stents, whereas AlPcS4 and MB are more suitable candidates as coating PSs. These results were further reconfirmed by thermogravimetric analysis (Fig. S5B to D). Upon heating from 30 to 300 °C, MB exhibited water removal (11.6%) at 98.8 °C and slight organic decomposition (2.1%) at 182.3 °C, whereas Ce6 showed organic decomposition (18.8%) at 190.7 °C. By contrast, AlPcS4 showed only water removal (13.2%) at 83.3 °C and no clear organic decomposition up to 300 °C. This confirms that AlPcS4 is the most stable PS at thermal drying conditions.

To evaluate ROS generation efficiency under repeated irradiation, DMA was used as a probe for singlet oxygen (^1^O_2_). The intrinsic fluorescence of DMA decreases upon reaction with ^1^O_2_, making it an appropriate indicator for the type II mechanism of ROS generation. Although all 3 PSs generated ROS in a light-dependent manner, MB induced only a slight decrease in DMA fluorescence (15.3% decreased after 90 J cm^−2^ irradiation), reflecting its inherently low photoconversion efficiency (Fig. 2B). Ce6, despite its strong photoconversion properties in organic solvents, aggregated within the silicone membrane, resulting in only a marginal improvement over MB (30.9% decrease after 90 J cm^−2^ irradiation). In contrast, AlPcS4 showed a distinct and stepwise decrease in DMA fluorescence over 3 irradiations (30 J cm^−2^ each), indicating continuous and effective ROS generation (67.2% decrease after 90 J cm^−2^ irradiation), confirming its stability and superior ROS generation ability under strong and repeated laser irradiations. In the SOSG assay conducted in aqueous solution at the same molar concentrations (0.1 μM), ROS generation was observed only in the AlPcS4 group (Fig. S7). This confirms that AlPcS4 is the most suitable PS for the stent-based catheter coating membrane.

### Chemical characterization of the AlPcS4-embedded stent-based catheter system

It is well known that π–π stacking between aggregated PSs can quench their excited states, thereby reducing ROS generation [33]. To prevent aggregation-induced quenching of AlPcS4 and ensure its stable photodynamic performance, we optimized its dispersion within silicone membranes and investigated its coordination interactions with the silicone membrane. XPS analysis was performed on both the pristine silicone membrane and the AlPcS4-embedded membrane (Fig. 2C and D and Figs. S8 and S9). In the AlPcS4-embedded membrane, distinct Al 2p and S 2p peaks were observed, with a reduced –SH peak at 164.9 eV after high-temperature baking while drying. Notably, the Al–O binding energy shifted from 74.4 eV (Al–N and Al–Cl) to 77.5 eV, consistent with the coordination between Al^3+^ ions and silanol (Si–OH) groups (O–Al–N) [34]. In the O 1s spectrum, the Si–OH peak at 532.3 eV also intensified due to overlap with the sulfonic acid (–SO_3_H) moiety. Hydrogen bonding of Si–OH to the sulfonate moiety intensifies the SiO_2_ peak at 532.4 eV. A minor Al–O peak at 530.6 eV further corroborated the Al–OH coordination. Following AlPcS4 incorporation, the C–H/C–C peaks at 284.9 eV grew stronger, and the N–Si peak at 401.5 eV merged with the N–Al peak at 400.9 eV to form a single enhanced band. These spectral shifts demonstrated that AlPcS4 was dispersed in the silicone membrane via coordination bonding between the Al^3+^ ion center and the membrane’s silanol groups. As PDT does not require the release of the PS [35], this coordination bonding reduces aggregation-induced quenching and enhances the photodynamic quantum yield.

### Phototoxicity of repeated PDT using the AlPcS4-embedded membrane

**Fig. 3.**
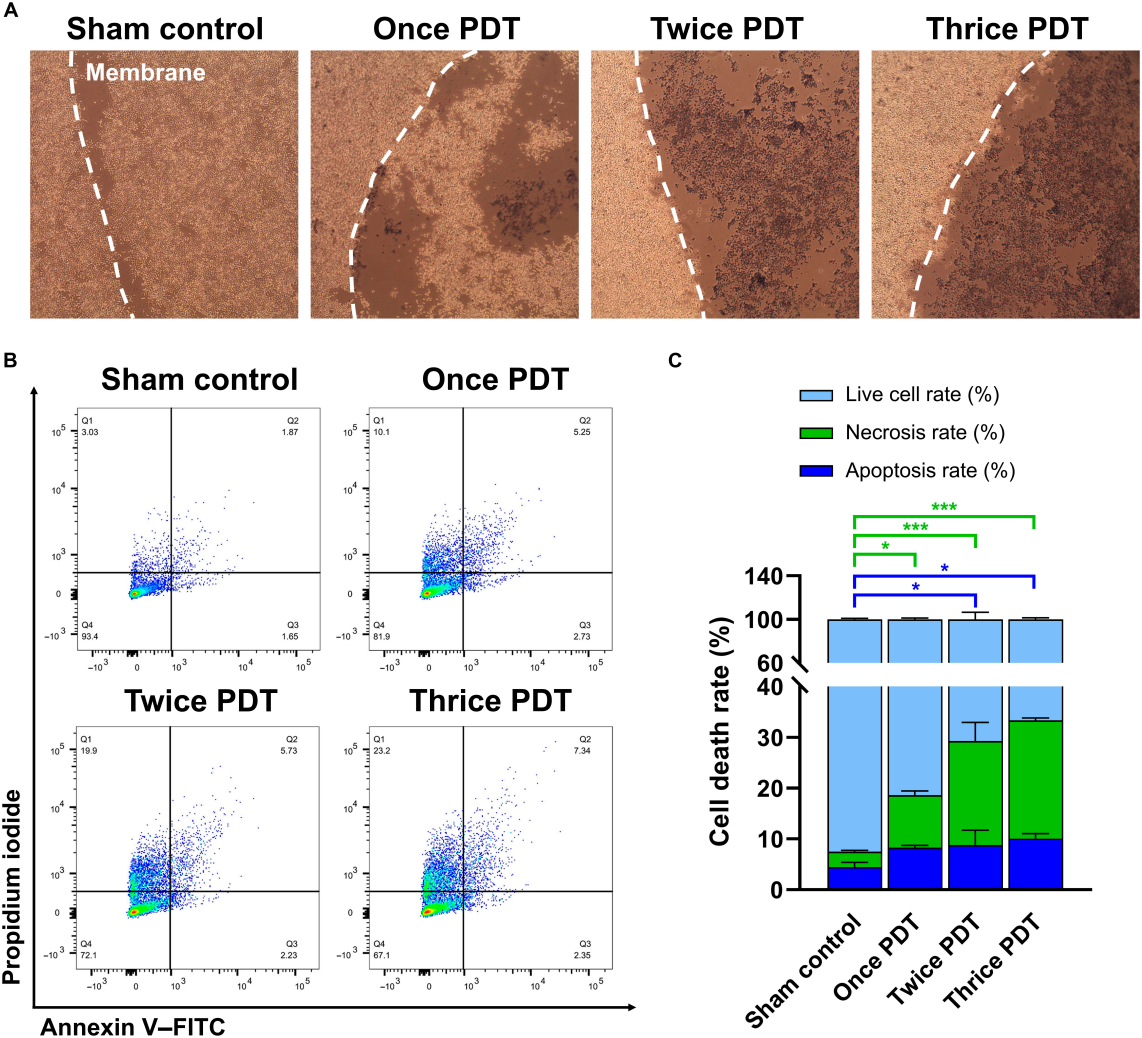
In vitro phototoxicity analysis of a photoactive membrane. (A) Phototoxicity analysis of repeated PDT using the AlPcS4-embedded membrane (white dashed line) with trypan blue staining. (B) Analysis of the apoptosis and necrosis induced by repeated PDT using flow cytometry with annexin V–FITC/propidium iodide staining. (C) Apoptosis and necrosis rates from repeated PDT analysis from (B). The power of the laser was 3 J cm^−2^ (50 mW cm^−2^ for 1 min) each time. Repeated PDT initially induces apoptosis-mediated cell death; however, with repetition, necrosis becomes the predominant cell death mechanism. Note: Data are presented as mean ± standard deviation. *P < 0.05; ***P < 0.001. FITC, fluorescein isothiocyanate.

To evaluate whether the AlPcS4-embedded membrane can induce cancer cell death under repeated irradiation, KYSE-70 cells were subjected to up to 3 consecutive PDT sessions at 3 J cm^−2^ each, followed by trypan blue staining and an apoptosis/necrosis detection assay. A previous study showed nearly complete cell death at an irradiation fluence of 5 J cm^−2^ [18]. Therefore, we lowered the intensity to 3 J cm^−2^ to examine PDT-number-dependent effects. Representative images showing the phototoxic effects of repeated PDT with the AlPcS4-embedded membrane are presented in Fig. 3A. No significant cell death staining was observed when the membrane was attached to the cells without irradiation. The once-PDT group produced only partial cell death in the membrane region, whereas the twice- and thrice-PDT groups achieved almost complete cell death in the same area. Thus, repeated PDT ensures reliable phototoxicity in esophageal cancer cells.

To compare cell death rates across repeated PDT sessions, apoptosis/necrosis (annexin V/propidium iodide)-stained cells were analyzed using flow cytometry (Fig. 3B and C). Q1 represented necrotic cells, while Q2 and Q3 represented late and early apoptotic cells, respectively. In the dot plots of the no-PDT group, most cells remained viable (Q4), with a few dead cells possibly resulting from mechanical handling during membrane attachment. As the PDT session progressed, the cell population progressively shifted into Q1, confirming necrosis as the dominant cell death pathway. Although the fraction of apoptotic cells increased slightly from the once- to the thrice-PDT cycle, the increase was modest. However, the necrotic fraction in the twice- and thrice-PDT groups was significantly higher than that in the once-PDT group. Considering that apoptosis and necrosis were initially observed at comparable levels after the first PDT session, this suggests that accumulated photodynamic damage may exceed the cellular repair capacity, shifting the cell death mechanism from programmed apoptosis to uncontrolled necrosis [36,37]. Considering the high metastatic potential of esophageal carcinoma and its tendency to develop resistance to apoptosis under repeated PDT, necrosis-driven local cell death and inflammation-mediated immunogenic effects may offer more therapeutic benefits. These findings demonstrate that the AlPcS4-embedded membrane is suitable for repeated PDT treatment of esophageal carcinoma.

### In vivo anticancer effect of repeated PDT using the AlPcS4-embedded membrane

**Fig. 4.**
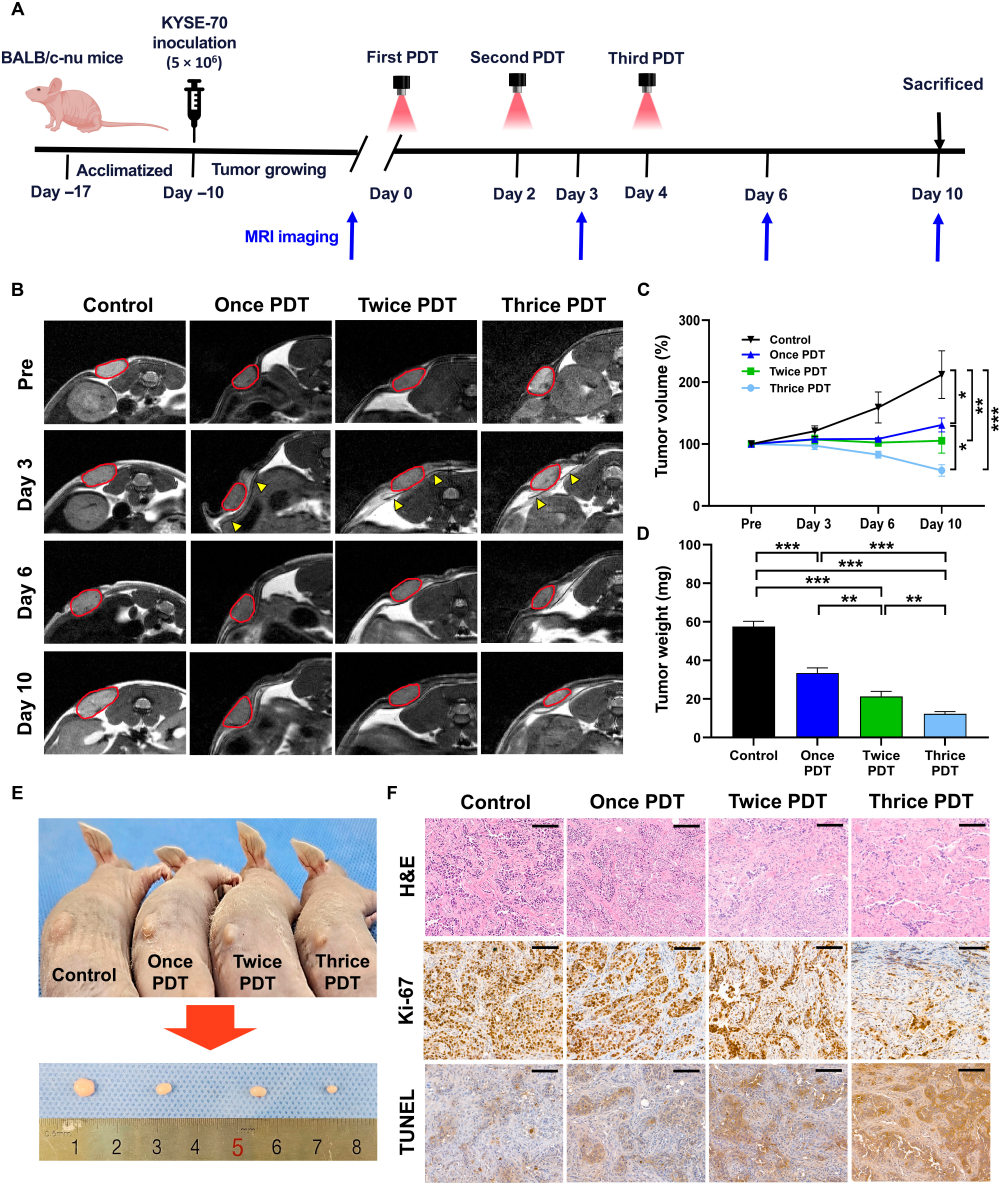
In vivo anticancer effect of repeated PDT using a photoactive membrane in the xenograft tumor model. (A) Schematic representation of the experimental design. (B) Representative axial T_2_-weighted MR images of a mouse with KYSE-70 tumor from each group over time. The yellow arrowheads on day 3 indicate the membrane. (C) Graph showing the percentage of tumor volume change. (D) Graph showing the extracted tumor weight. (E) Representative mouse of each group and the extracted tumor. (F) Representative microscopic images of hematoxylin and eosin-, Ki-67-, and TUNEL-stained tissue slides (scale bars: 100 μm). Repeated PDT using the AlPcS4-embedded membrane effectively suppressed tumor growth and promoted cell death in a treatment-number-dependent manner. Note: Data are presented as mean ± standard deviation. *P < 0.05; **P < 0.01; ***P < 0.001. MR, magnetic resonance; TUNEL, terminal deoxynucleotidyl transferase-mediated dUTP nick end labeling; H&E, hematoxylin and eosin.

KYSE-70 cells were inoculated into BALB/c nude mice to establish a tumor model and investigate the anticancer efficacy of repeated PDT. The detailed schedule of the xenograft experiment is indicated in Fig. 4A. The AlPcS4-embedded membrane was successfully inserted under the tumor in all mice designated for PDT, without any procedure-related complications. The PDT-treated mice survived until the end of the study. The body weights in the PDT-treated groups tended to decrease in proportion to the number of repetitions (Fig. S10).

The representative MR images and changes in tumor volume are shown in Fig. 4B and C. Tumor volume changes (%) on day 10 are summarized in Table S1. On day 10, the mean percentage of tumor volume changes differed significantly between the groups (all variables; *P* < 0.001, one-way ANOVA). The mean tumor volume changes in the once-PDT (*P* = 0.002) and twice-PDT groups (*P* = 0.011) were significantly lower than that in the control group. Furthermore, the tumor volume changes in the thrice-PDT group were also significantly lower than those in the control and once-PDT groups (*P* < 0.001 and *P* = 0.018). In the MR images of all membrane-inserted mice, the membrane remained stably positioned under the tumor until sacrifice, as indicated by the yellow arrowheads in Fig. 4B.

All mice were euthanized for gross and histological examinations. The tumors were successfully harvested, and the weight of each tumor was measured (Fig. 4D and E). Tumor weight on day 10 is summarized in Table S1. The mean tumor weight differed significantly between the groups (all variables; *P* < 0.001, one-way ANOVA). The tumor weights in the once-, twice-, and thrice-PDT groups were significantly lower than those in the control group (all *P* < 0.001). In addition, the twice-PDT and thrice-PDT groups showed significantly lower tumor weights than the once-PDT group (*P* = 0.001 and *P* < 0.001), and the thrice-PDT group exhibited a significantly lower tumor weight than the twice-PDT group (*P* = 0.007).

An anticancer effect of PDT using the AlPcS4-embedded membrane was evident from the significant reductions in tumor volume changes and weight on day 10 in the PDT-treated groups. The tumor suppression effect increased proportionally with the number of PDT sessions, with the thrice-PDT group showing the most pronounced reduction in tumor size, indicating that repeated and periodic irradiation plays a critical role in sustaining therapeutic efficacy. These findings are consistent with those of previous studies demonstrating that multiple PDT exposures can effectively overcome tumor cell repair mechanisms and improve overall therapeutic outcomes [38,39]. Our data further support this concept, suggesting that cumulative oxidative stress induced by repeated PDT surpasses the cellular threshold for repair, thereby leading to durable tumor regression. Interestingly, unlike the growth suppression observed in previous studies employing 3 sessions of PDT, our approach resulted in substantial tumor ablation, with the tumor volume reduced to 57.40% ± 9.26% compared to the baseline. This enhanced therapeutic effect is presumably attributed to the implantation of the AlPcS4-embedded membrane directly beneath the tumor, which maintains photodynamic activity, photostability, and high quantum yield under periodic and repeated irradiation, compared to conventional intravenous PS administration. Taken together, these results highlight the potential of the AlPcS4-embedded membrane as a promising candidate for periodic and repeated PDT and provide supporting evidence for its catheter-based application in subsequent large-animal studies.

### Histological findings

The results of histological evaluations are shown in Fig. 4F. H&E staining revealed a gradual increase in disrupted tumor architecture, inflammatory infiltrations, and interstitial spaces with repetitive PDT, whereas compactly arranged tumor cells with intact cell morphology were observed in the control group. Ki-67 staining showed a reduction in tumor cell proliferation that correlated with the number of PDT sessions. Similarly, TUNEL staining demonstrated an increase in apoptotic cell deposition according to the number of PDT sessions, with the thrice-PDT group exhibiting the highest apoptotic activity. Furthermore, based on the increased TUNEL-positive staining and the prominent inflammatory cell infiltration observed in H&E staining, necrotic cell death is also presumed to have been induced in the thrice-PDT group.

Tumors show unusual proliferation, differentiation, and abnormal apoptosis. Inhibiting the proliferation of tumor cells and enhancing apoptosis are basic mechanisms of action of many therapeutic modalities [40]. Ki-67 is a nuclear antigen widely used as a marker of cell proliferation. Recent studies have shown that aluminum-phthalocyanine chloride tetrasulfonate-mediated PDT induces cell cycle arrest at the G0/G1 phase in esophageal cancer cells via down-regulation of Ki-67 expression, thereby suppressing cellular proliferation [41]. Consistent with the results of a prior report, our AlPcS4-based PDT also demonstrated a tumor-suppressive effect, as evidenced by a reduction in Ki-67-positive staining, which was further enhanced with repeated treatment. Furthermore, repeated PDT induced notable apoptosis in tumor tissues, as demonstrated by a treatment-dependent increase in TUNEL-positive staining. These results suggest that repeated and periodic PDT using an AlPcS4-embedded membrane can markedly suppress proliferative activity and enhance cell death in esophageal carcinoma cells.

### Procedural outcomes of repeated PDT in the porcine esophagus

**Fig. 5.**
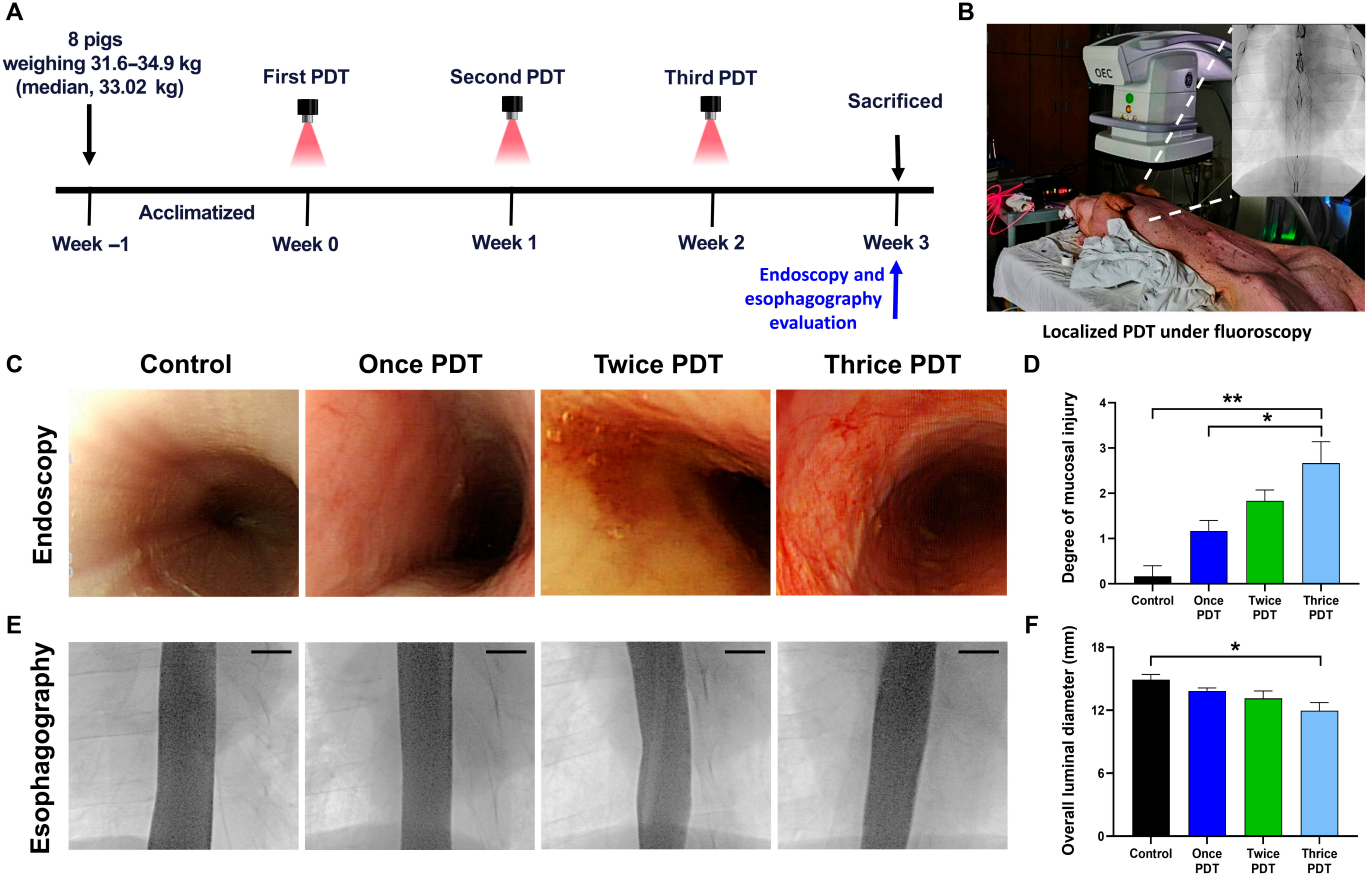
Study design and follow-up endoscopic and esophagographic findings. (A) Schematic representation of the animal study design. (B) Photograph showing the localized PDT administration under fluoroscopic guidance in a porcine model. (C) Representative photographs of endoscopic findings in the control and repeated-PDT-treated groups are presented, (D) together with a graph quantifying the degree of mucosal injury. (E) Representative photographs of esophagography findings in the control and repeated-PDT-treated groups are presented (scale bars: 10 mm), (F) together with a graph quantifying the degree of overall luminal diameter. Mucosal injury progressively worsened with repeated PDT sessions, accompanied by modest stricture formation. Note: Data are presented as mean ± standard deviation. *P < 0.05; **P < 0.01.

Previously, a PS-embedded stent-based catheter has been successfully applied to rat colon and rabbit esophagus models [17,18]. In the present study, the catheter was scaled up and optimized to evaluate the possibility of translation from the bench to the clinic. The detailed schedule of the porcine experiment and representative PDT procedure photographs are shown in Fig. 5A and B. We found that localized PDT using the AlPcS4-embedded stent-based catheter was technically successful in all pigs without any procedure-related complications. Body weight tended to increase more slowly with a higher number of treatment repetitions, presumably due to the pain associated with mucosal injury (Fig. S11). Despite recent advances in nanomaterials for PDT, clinical translation remains challenging due to unresolved issues, including limited tissue penetration depth, heterogeneous intratumoral distribution of PSs, and difficulties in achieving precise control of light delivery in tissue [42]. Previously, PDT using a PS-embedded membrane-covered stent was developed to relieve esophageal strictures via the delivery of singlet oxygen to the mucosal surface [14]. This approach induced sufficient cytotoxic effects in tissues in contact with the membrane and enabled localized PDT with a single esophageal access, eliminating the need for systemic PS administration and the 40- to 48-h postinjection waiting period required in conventional PDT protocols. However, it has also been associated with stent-related complications such as restenosis and migration and uneven light delivery due to the noncentral positioning of the flimsy fiber. To overcome these technical challenges, we developed a retrievable PS-embedded stent-based catheter system that enables safe removal immediately after PDT without stent-related complications. In addition, a cylindrical fiber can be inserted through the inner tube and centrally positioned within the stent lumen to enable precise and efficient light delivery during PDT. It simplifies the PDT procedure and enhances safety, providing an alternative treatment option for the elderly or surgically ineligible patients with progressive esophageal carcinoma. Furthermore, in this study, we integrated catheter-based technology with repeated AlPcS4-mediated irradiation, which enhanced therapeutic depth, promoted more uniform PS distribution, and enabled precise light delivery, thereby offering a strategy to overcome the major limitations of conventional PDT. With growing clinical interest and supportive data, we believe that it may represent a promising novel approach for treating endoluminal malignancies in nonvascular organs such as the gastroduodenum, colon, bile duct, and urethra.

### Endoscopic and esophagographic findings

Endoscopy and esophagography were successfully performed after the PDT procedure. The endoscopic and esophagographic findings are summarized in Table S2, and the representative images are shown in Fig. 5C and E. Mild mucosal hyperemia was observed in the once-PDT group. The twice-group exhibited yellowish scab-like lesions, suggesting tissue healing after mucosal injury. In the thrice-PDT group, scattered hemorrhages were prominently observed, indicating a transition to moderate injury. The mean degree of mucosal injury differed significantly between the groups (all variables; *P* = 0.006, one-way ANOVA). The thrice-PDT group showed a significantly higher mucosal injury score than the control group and once-group (*P* = 0.005 and *P* = 0.02). The overall luminal diameter gradually decreased with an increase in the number of treatment sessions. The mean overall luminal diameter differed significantly between the groups (all variables; *P* = 0.033, one-way ANOVA). The thrice-PDT group exhibited a significantly lower diameter than the control group (*P* = 0.027). Endoscopic and esophagographic evaluation demonstrated that mucosal injury progressively worsened with an increase in the number of treatment sessions, accompanied by slight stricture formation.

Endoscopic submucosal dissection is widely accepted as a standard treatment for esophageal carcinoma [43,44]. However, circumferential resections over 5 cm in length in the diffused long segment are often avoided due to the high risk of persistent stricture formation [45,46]. PDT has been proposed as an alternative treatment for diffused long-segment or whole-circumferential esophageal carcinoma; however, lesions exceeding 10 cm in length have still been considered a major risk factor [47]. In this study, the repeatable AlPcS4-embedded stent-based catheter system demonstrated potential as a useful approach for circumferential and diffused esophageal lesions longer than 10 cm. Owing to the high photostability and minimal photobleaching properties of AlPcS4, repeated applications within a single session are feasible even for lesions longer than 10 cm. In this study, the fabricated AlPcS4-embedded catheter system successfully induced localized mucosal injury without procedure-related complications in all porcine esophagus models, which are anatomically similar to those of humans. Thus, the repeatable AlPcS4-embedded stent-based catheter system demonstrates considerable clinical potential as a promising therapeutic option for esophageal carcinoma, particularly in cases involving circumferential or diffused lesions exceeding 10 cm.

### Histological findings

**Fig. 6.**
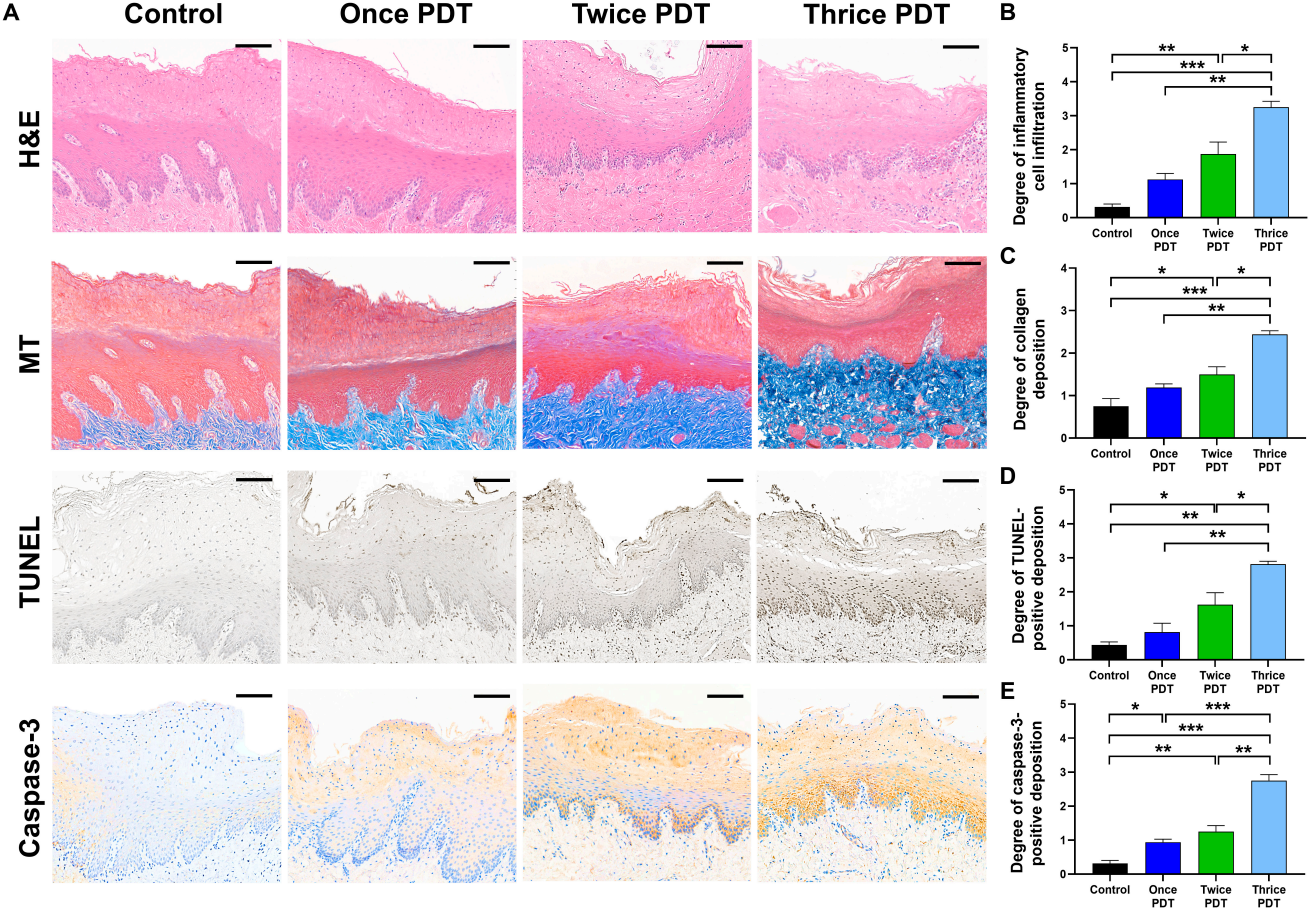
Histological findings in control and repeated-PDT-treated groups with a photoactive stent-based catheter in the porcine esophageal model. (A) Representative microscopic images of hematoxylin-and-eosin-, Masson’s trichrome-, TUNEL-, and caspase-3-stained tissue slides are shown (scale bars: 100 μm). (B to E) Histological results of the control and repeated-PDT-treated groups regarding the degree of (B) inflammatory cell infiltration, (C) collagen deposition, (D) TUNEL-positive deposition, and (E) caspase-3 positive deposition. The levels of inflammation, fibrosis, and apoptosis markers increased with repeated PDT sessions. Note: Data are represented as mean ± standard deviation. *P < 0.05; **P < 0.01; ***P < 0.001. MT, Masson’s trichrome.

The entire esophagus of pigs was successfully extracted at the endpoint of the study. The histological findings are summarized in Table S3, and representative images are shown in Fig. 6A. The mean degrees of inflammatory cell infiltration, collagen deposition, TUNEL-positive deposition, and caspase-3-positive deposition differed significantly between the groups (*P* < 0.001, *P* = 0.001, *P* = 0.002, and *P* < 0.001, respectively; one-way ANOVA). Histological analyses revealed that the degrees of inflammatory cell infiltration, collagen deposition, TUNEL-positive staining, and caspase-3-positive staining progressively increased with the number of PDT sessions. Quantitative assessments showed that the thrice-PDT group exhibited the highest levels for all parameters compared to the control and once-PDT groups, while the twice-PDT group showed an intermediate increase (Fig. 6B to E). The progressive increase in inflammatory infiltration and fibrosis observed in the esophagus suggests that repeated PDT induced cumulative tissue damage. This finding is consistent with those of previous studies demonstrating that repeated tissue injury worsened local inflammation and fibrotic remodeling [48]. The depth of TUNEL- and caspase-3-positive staining increased with the number of PDT sessions, indicating that repeated PDT produces ROS that can reach into the submucosal layer, where esophageal carcinoma typically invades. These findings support the hypothesis that repeatable AlPcS4-embedded stent-based PDT enhances therapeutic efficacy by enabling deeper and more sustained ROS-mediated cytotoxicity compared to single-session PDT.

Esophageal carcinoma is primarily localized to the mucosal and submucosal layers, requiring treatment modalities that ensure selective tumor removal while preserving the structural and functional integrity of the outer esophageal wall [49]. As minimally invasive modalities for the local treatment of esophageal cancer, stent-based techniques such as radio-frequency ablation and irreversible electroporation have been developed [7,8]. However, their clinical use has been limited due to the risk of thermal injury beyond the submucosal layer and the esophagus’s anatomical adjacency to the heart, which may result in complications such as arrhythmias [9,10]. In contrast, our localized PDT approach generates ROS with a short lifespan and lower tissue penetration, providing a safer and effective therapeutic option for esophageal carcinoma. However, the shallow penetration of ROS may result in rapid tissue recovery within 2 to 4 weeks, raising concerns regarding potential recurrence [15–18]. Histological analysis in the porcine model revealed a progressive increase in the depth of cellular destruction with repeated PDT sessions. Notably, after 3 exposures, substantial apoptotic cell death extended into the submucosal layer without any adverse effects while preserving the overall esophageal structure. These findings suggest that periodic and repeated PDT may act as a safe and effective therapeutic strategy for esophageal carcinomas requiring precise control over treatment depth.

Although many of the variables of interest reached statistical significance, the sample size was too small to perform a robust statistical analysis. In addition, the sacrifice timepoint based on the first PDT created different posttreatment intervals between the study groups, which may have influenced the outcomes. Furthermore, some of the measurements in this study were obtained subjectively, and quantitative data such as cell number would have provided more reliable data. Although further studies with large sample sizes are required to confirm its safety and efficacy, the present study provided a novel strategy for the local treatment of esophageal carcinoma to enhance and maintain therapeutic efficacy.

## Conclusion

A repeatable photoreactive stent-based catheter represents an innovative therapeutic strategy for esophageal carcinoma. This system takes advantage of the excellent photostability, anti-photobleaching, and high quantum yield properties of AlPcS4 while addressing its low delivery efficiency via local embedding into the stent membrane. Intracellular analysis revealed that repeated PDT primarily induced necrotic cell death, suggesting its potential to overcome apoptosis resistance in esophageal carcinoma. Experiments in a xenograft tumor model have confirmed the superior therapeutic efficacy of the repeated PDT using an AlPcS4-embedded membrane, as evidenced by significant tumor regression along with extensive apoptotic and necrotic cell death. In vivo trials in a porcine esophagus model demonstrated that repeated and periodic PDT using an AlPcS4-embedded stent-based catheter was technically safe and facilitated ROS penetration across the submucosal layer where esophageal carcinoma primarily invades while also maintaining therapeutic efficacy throughout the treatment period. Taken together, our findings from both xenograft and porcine models demonstrate that the repeatable AlPcS4-embedded stent-based catheter can safely induce cumulative cell death and holds clinical promise as a therapeutic option for esophageal carcinoma. This therapeutic strategy can also be applied to esophageal carcinoma with circumferential or diffuse lesions longer than 10 cm, based on the repeatable activation properties of AlPcS4, to enhance treatment efficacy.

## Data Availability

The datasets used and/or analyzed during the current study are available from the corresponding authors on reasonable request.
