# Supplementary material for: Repeatable Photoactive Stent-Based Catheter to Enhance Therapeutic Efficacy for Esophageal Carcinoma
Source: Biomater Res. 2025 Oct 30;29:0274. doi: 10.34133/bmr.0274 (PMC12697060; doi:10.34133/bmr.0274)
Supplement: Supplementary 1 — Figs. S1 to S11 Tables S1 to S3 [file bmr.0274.f1.zip › [BMR] Supporting Information.docx]

**Repeatable Photoactive Stent-based Catheter to Enhance Therapeutic Efficacy for Esophageal Carcinoma**

Seung Jin Eo^1,2^†, Hyeonseung Lee^3^†, Ji Won Kim^1,2^, Song Hee Kim^1,2^, Dong-Sung Won^1,2^, Yubeen Park^2^, Kun Na^3*^, Do Hoon Kim^4*^, Jung-Hoon Park^1, 2*^

**Affiliations**

^1^ Department of Convergence Medicine, Asan Medical Center, University of Ulsan College of Medicine, 88 Olympic-ro 43-gil, Songpa-gu, Seoul, 05505, Republic of Korea

^2^ Biomedical Engineering Research Center, Asan Institute for Life Sciences, Asan Medical Center, 88 Olympic-ro 43-gil, Songpa-gu, Seoul, 05505, Republic of Korea

^3^ Department of Biotechnology, Department of Biomedical-Chemical Engineering, The Catholic University of Korea, 43 Jibong-ro, Wonmi-gu, Bucheon-si, Gyeonggi-do, 14662, Republic of Korea

^4^ Department of Gastroenterology, Asan Medical Center, University of Ulsan College of Medicine, 88 Olympic-ro 43-gil, Songpa-gu, Seoul, 05505, Republic of Korea

Address correspondence to: Kun Na; kna6997@catholic.ac.kr, Do Hoon Kim; kdh@amc.seoul.kr, and Jung-Hoon Park; [jhparkz@amc.seoul.kr](mailto:jhparkz@amc.seoul.kr)

^*^ K.N., D.H.K., and J.-H.P. contributed equally to this work and are the co-corresponding authors.


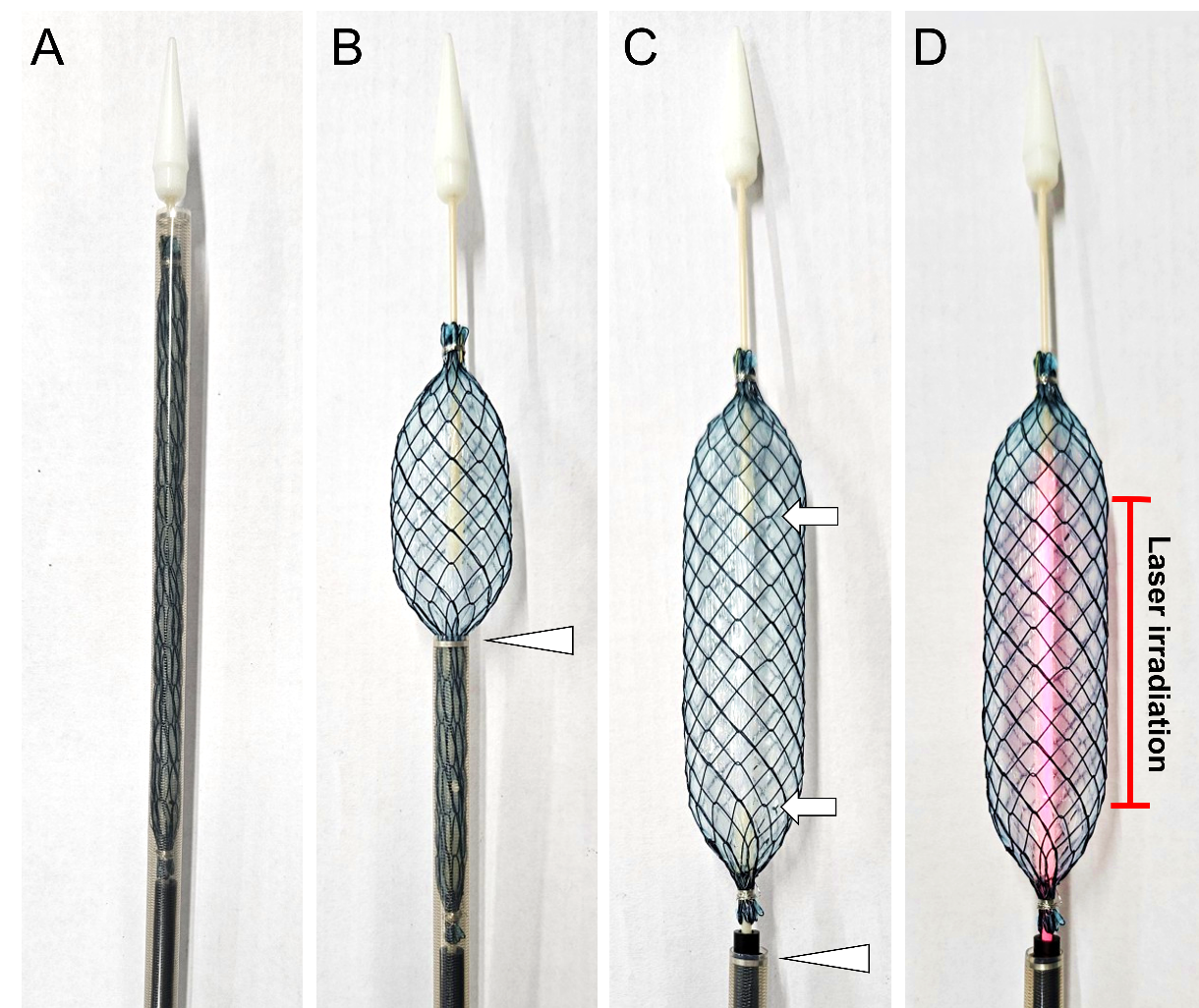


**Figure S1.** Photographs showing the photoactive stent-based catheter to perform localized PDT. A) The AlPcS4-embedded stent is loaded into the catheter system. B) The stent is deployed by pulling the braided tube (arrowhead). C) After full deployment, a cylindrical fiber (white arrows) was inserted into the middle portion of the AlPcS4-embedded stent. D) PDT was performed under laser irradiation. Note: AlPcS4, aluminum (III) phthalocyanine chloride tetrasulfonic acid; PDT, photodynamic therapy.


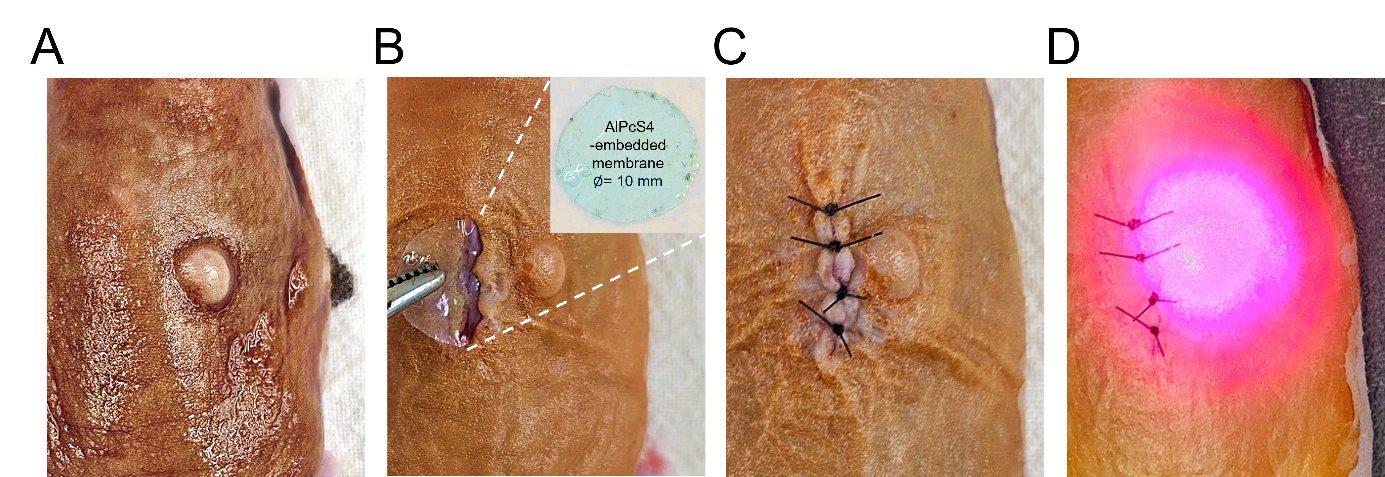


**Figure S2.** Photographs showing procedural steps for localized PDT using photoactive membrane in the xenograft tumor model. A) Povidone was applied to the mouse model to ensure disinfection. B) An incision was made and the AlPcS4-embedded membrane was inserted under the tumor. C) Sutured and D) repeated PDT treatment was performed with the assigned number of laser irradiations. Note: AlPcS4, aluminum (III) phthalocyanine chloride tetrasulfonic acid; PDT, photodynamic therapy.


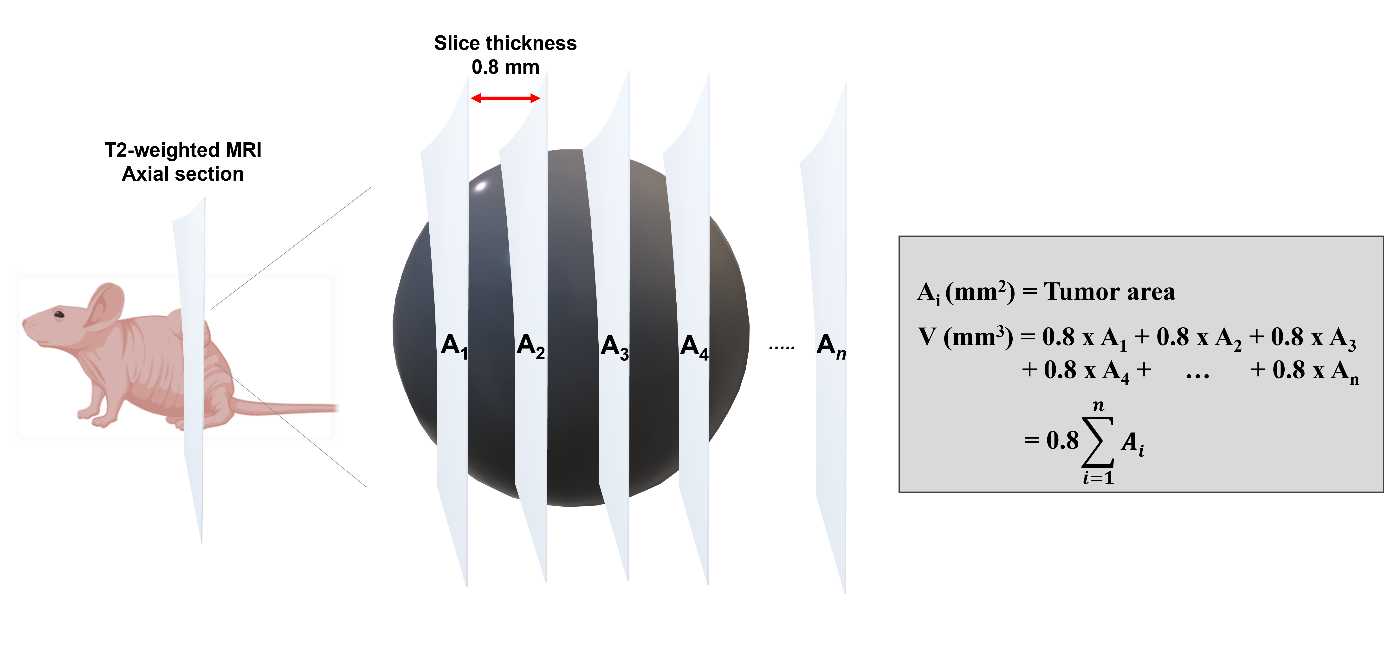


**Figure S3.** Schematic illustration showing ROI-based volume calculation methods from MRI. Note: ROI, region of interest; MRI, magnetic resonance imaging.


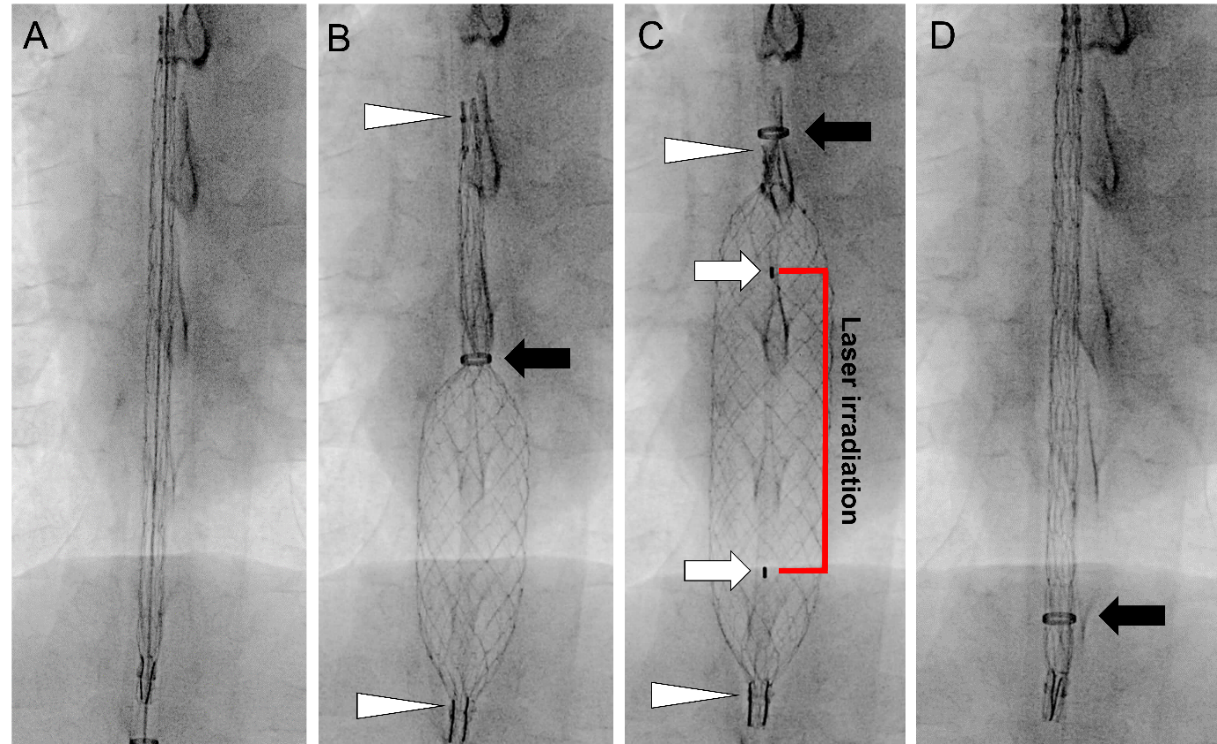


**Figure S4.** Radiographic images showing the technical steps for localized PDT using a photoactive stent-based catheter in the porcine esophageal model. A) A 0.035-inch guidewire was advanced through the esophagus into the stomach and the AlPcS4-embedded stent-based catheter was inserted over the guidewire. B) The AlPcS4-embedded stent (white arrowheads) was deployed by pulling the braided tube (black arrow). C) The cylindrical fiber (white arrows) was inserted into the middle portion of the stent (arrowheads) through the distal port of the catheter. D) After the PDT, the fiber was removed and the expanded stent was recaptured by advancing the braided tube (black arrow); the catheter system was smoothly removed. Note: AlPcS4, aluminum (III) phthalocyanine chloride tetrasulfonic acid; PDT, photodynamic therapy.

*^
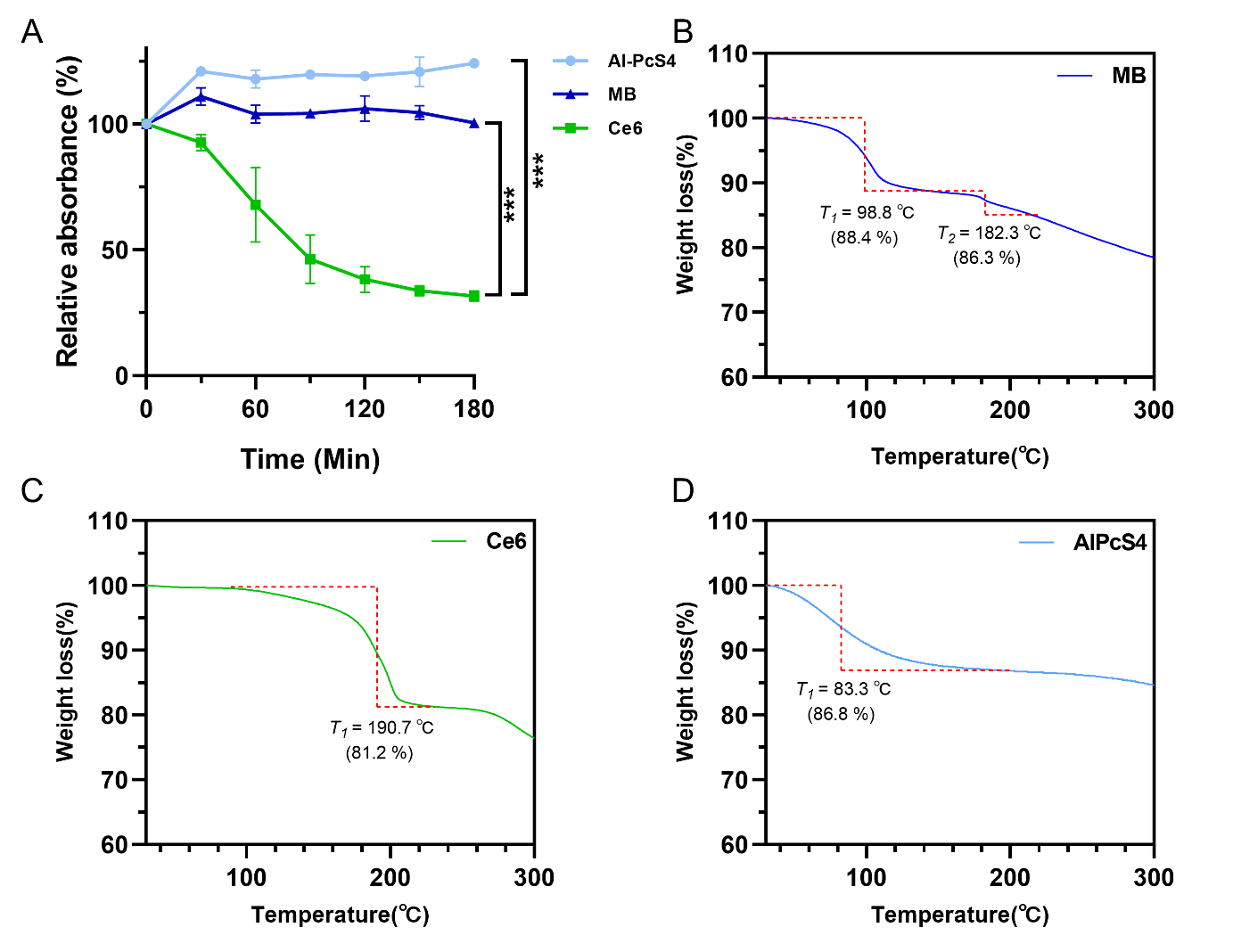
^*

**Figure S5.** PSs degradation under drying condition (150 ℃) at various time points. A) Relative UV-Vis spectroscopic peak absorbance (%) of each PS at various exposure time at 150 ℃. B-D) TGA spectrum of B) MB, C) Ce6, and D) AlPcS4 at 10 ℃ min^-1^ ramping rate for 300 ℃ under N_2_ atmosphere. *T_1_* and *T_2_* indicates inflection-point temperature with residual mass below. Note: AlPcS4, aluminum (III) phthalocyanine chloride tetrasulfonic acid; MB, methylene blue; Ce6, chlorin e6, PS, photosensitizer, TGA, Thermogravimetric Analysis.

**Figure S6.** Graph showing the film thicknesses of various PSs embedded silicone membranes. Note: AlPcS4, aluminum (III) phthalocyanine chloride tetrasulfonic acid; MB, methylene blue; Ce6, chlorin e6; PS, photosensitizer.

**Figure S7.** SOSG generation analysis with various PSs (0.1 μM) embedded film when laser irradiated (100 mW cm-2, 50s, 6 times repeated). Fluorescence of SOSG (Excitation: 504 nm, Emission: 525 nm) was interpreted as ROS generation. Note: SOSG, Singlet oxygen sensor green; AlPcS4, aluminum (III) phthalocyanine chloride tetrasulfonic acid; MB, methylene blue; Ce6, chlorin e6; PS, photosensitizer.


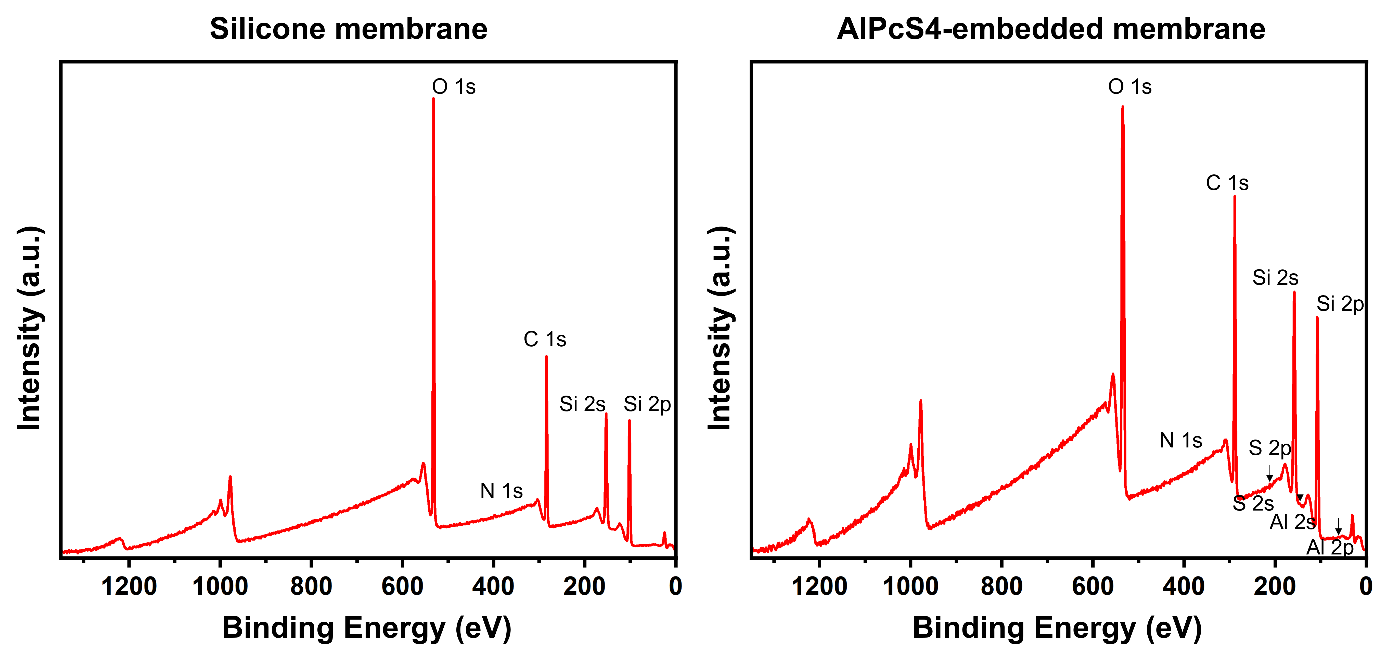


**Figure S8.** Total XPS spectrum of silicone membrane and AlPcS4-embedded membrane. Note: AlPcS4, aluminum (III) phthalocyanine chloride tetrasulfonic acid; XPS, X-ray photoelectron spectroscopy.


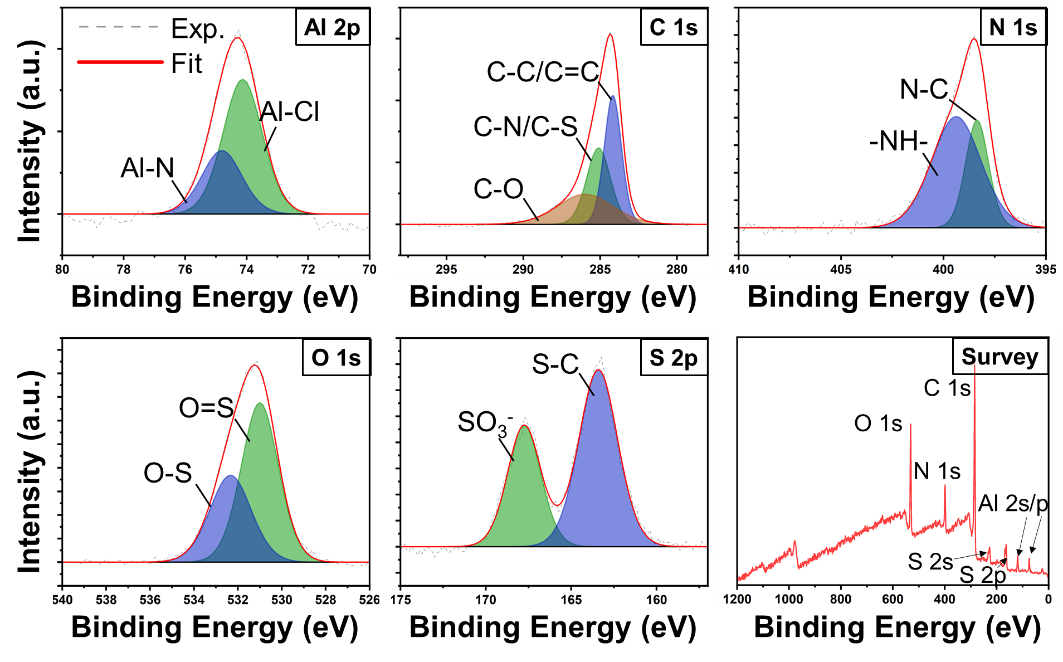


**Figure S9**. Narrow and survey XPS spectrum of the AlPcS4 powder at various element peaks (Al 2p, C 1s, N 1s, O 1s, S 2p and survey spectrum). Note: XPS, X-ray photoelectron spectroscopy; AlPcS4, aluminum (III) phthalocyanine chloride tetrasulfonic acid.


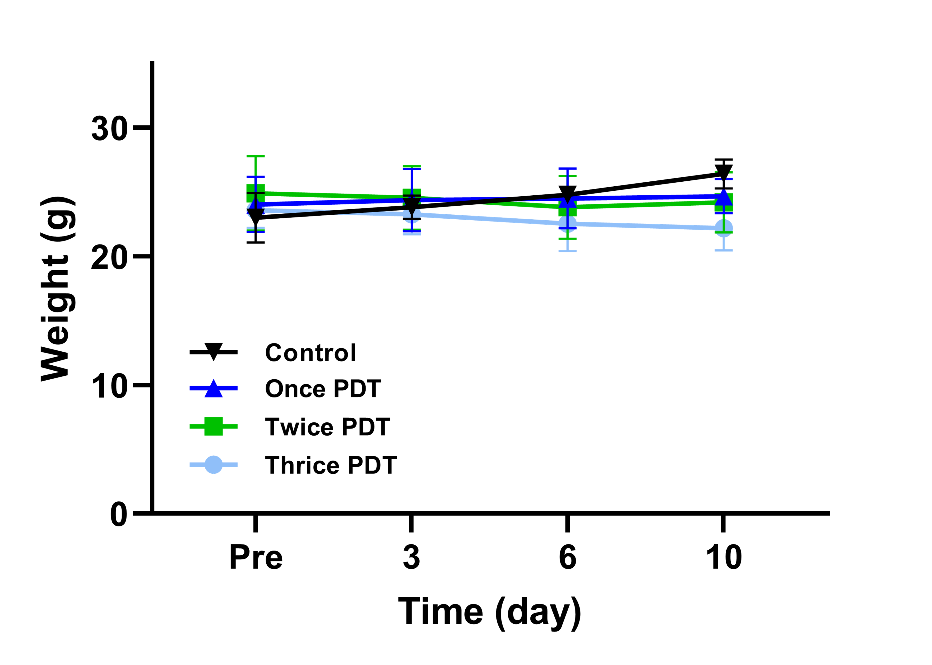


**Figure S10.** Body weight changes of the xenograft tumor model.


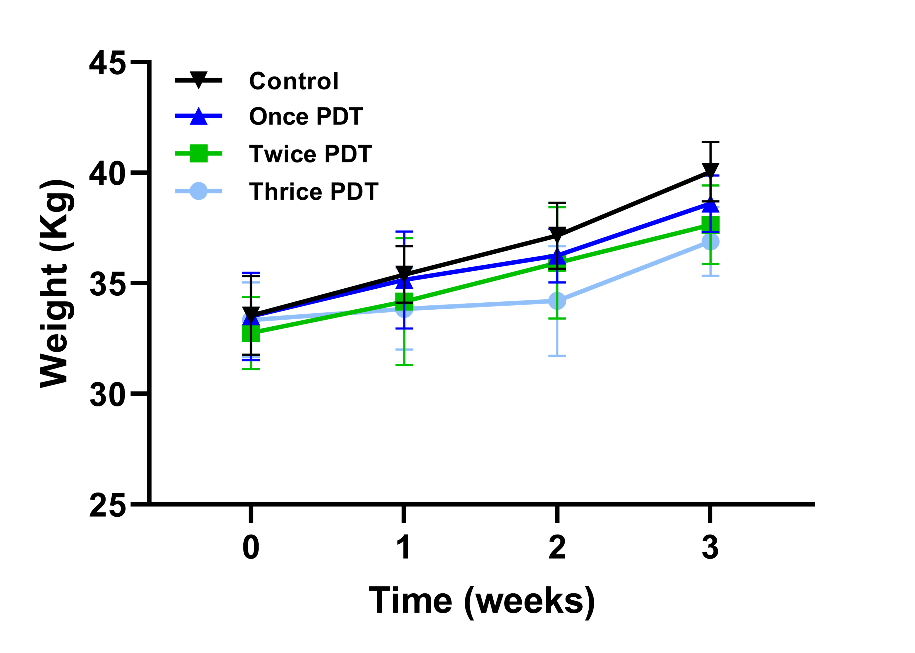


**Figure S11.** Body weight changes after the PDT procedure in enrolled pigs. Note: PDT, photodynamic therapy.

**Table S1.** Findings from the xenograft tumor model in all groups.

Data are presented as mean ± standard deviations. *One-way ANOVA with Tukey’s post hoc test.

|  | **Groups** | | | | **p-value*** | **p-value** | | | | | | |
| --- | --- | --- | --- | --- | --- | --- | --- | --- | --- | --- | --- | --- |
|  | ^A^ Control | ^B^ Once PDT | ^C^ Twice PDT | ^D^ Thrice PDT |  | A vs. B | A vs. C | A vs. D | B vs. C | B vs. D | C vs. D |  |
| Tumor volume changes in day 10 (%) | 212.07 ± 38.44 | 130.77 ± 11.25 | 105.47 ± 20.18 | 57.40 ± 9.26 | < 0.001 | 0.011 | 0.002 | < 0.001 | 0.558 | 0.018 | 0.122 |  |
| Tumor weight  (mg) | 57.57 ± 2.67 | 33.47 ± 2.65 | 21.30 ± 2.63 | 12.27 ± 1.21 | < 0.001 | < 0.001 | < 0.001 | < 0.001 | 0.001 | < 0.001 | 0.007 |  |

**Table S2.** Endoscopic and esophagographic findings in all groups of pigs.

|  | **Groups** | | | | **p-value*** | **p-value** | | | | | | |
| --- | --- | --- | --- | --- | --- | --- | --- | --- | --- | --- | --- | --- |
|  | ^A^ Control | ^B^ Once PDT | ^C^ Twice PDT | ^D^ Thrice PDT |  | A vs. B | A vs. C | A vs. D | B vs. C | B vs. D | C vs. D |  |
| Mucosal injury  (degree) | 0.165 ± 0.23 | 1.165 ± 0.23 | 1.663 ± 0.33 | 2.543 ± 0.39 | 0.006 | 0.104 | 0.020 | 0.005 | 0.285 | 0.029 | 0.169 |  |
| Overall luminal diameter (mm) | 14.895 ± 0.51 | 13.8 ± 0.28 | 12.93 ± 0.57 | 12.16 ± 0.66 | 0.033 | 0.383 | 0.127 | 0.027 | 0.677 | 0.117 | 0.351 |  |

Data are presented as mean ± standard deviations. *One-way ANOVA with Tukey’s post hoc test.

**Table S3.** Histological findings in all groups of pigs.

|  | **Groups** | | | | **p-value*** | **p-value** | | | | | |
| --- | --- | --- | --- | --- | --- | --- | --- | --- | --- | --- | --- |
|  | ^A^ Control | ^B^ Once PDT | ^C^ Twice PDT | ^D^ Thrice PDT |  | A vs. B | A vs. C | A vs. D | B vs. C | B vs. D | C vs. D |
| Inflammatory cell (degree) | 0.31 ± 0.09 | 1.12 ± 0.18 | 1.88 ± 0.35 | 3.25 ± 0.18 | < 0.001 | 0.069 | 0.007 | < 0.001 | 0.088 | 0.002 | 0.012 |
| Collagen (degree) | 0.75 ± .018 | 1.19 ± 0.09 | 1.5 ± 0.18 | 2.44 ± 0.09 | 0.001 | 0.111 | 0.020 | < 0.001 | 0.256 | 0.003 | 0.009 |
| TUNEL (degree) | 0.44 ± 0.09 | 0.81 ± 0.27 | 1.62 ± 0.35 | 2.81 ± 0.09 | 0.002 | 0.455 | 0.022 | 0.002 | 0.077 | 0.003 | 0.022 |
| Caspase-3 (degree) | 0.31 ± 0.09 | 0.94 ± 0.09 | 1.25 ± 0.18 | 2.75 ± 0.18 | < 0.001 | 0.037 | 0.009 | < 0.001 | 0.256 | <0.001 | 0.001 |

Data are presented as mean ± standard deviations. *One-way ANOVA with Tukey’s post hoc test. TUNEL: terminal deoxynucleotidyl transferase-mediated dUTP nick and labeling.
